# CP26 is not involved in qE- or qZ-type non-photochemical quenching in Arabidopsis

**DOI:** 10.1093/plphys/kiag207

**Published:** 2026-04-16

**Authors:** Julia Walter, Dhruv Patel-Tupper, Lam Lam, Alexa Ma, Georgia Taylor, Alistair Leverett, Graham R Fleming, Krishna K Niyogi, Johannes Kromdijk

**Affiliations:** Department of Plant Sciences, University of Cambridge, CB2 3EA Cambridge, United Kingdom; Department of Plant and Microbial Biology, University of California, Berkeley, CA 94720, United States; Howard Hughes Medical Institute, University of California, Berkeley, CA 94720, United States; Graduate Group in Biophysics, University of California, Berkeley, CA 94720, United States; Molecular Biophysics and Integrated Bioimaging Division, Lawrence Berkeley National Laboratory, Berkeley, CA 94720, United States; Department of Chemistry, University of California, Berkeley, CA 94720, United States; Department of Plant Sciences, University of Cambridge, CB2 3EA Cambridge, United Kingdom; Department of Plant Sciences, University of Cambridge, CB2 3EA Cambridge, United Kingdom; Graduate Group in Biophysics, University of California, Berkeley, CA 94720, United States; Molecular Biophysics and Integrated Bioimaging Division, Lawrence Berkeley National Laboratory, Berkeley, CA 94720, United States; Department of Chemistry, University of California, Berkeley, CA 94720, United States; Kavli Energy Nanoscience Institute, University of California, Berkeley, CA 94720, United States; Department of Plant and Microbial Biology, University of California, Berkeley, CA 94720, United States; Howard Hughes Medical Institute, University of California, Berkeley, CA 94720, United States; Molecular Biophysics and Integrated Bioimaging Division, Lawrence Berkeley National Laboratory, Berkeley, CA 94720, United States; Innovative Genomics Institute, University of California, Berkeley, CA 94720, United States; Department of Plant Sciences, University of Cambridge, CB2 3EA Cambridge, United Kingdom; Carl R Woese Institute for Genomic Biology, University of Illinois, 1206 W Gregory Drive, Urbana, IL IL61801, United States

## Abstract

CP26 is a monomeric minor light-harvesting complex of Photosystem II (LHCII) protein located at the interface between LHCII trimers and the PSII core in thylakoid membranes. Previous studies have proposed that CP26 plays a role in non-photochemical quenching (NPQ) in addition to light harvesting. Here, we utilized biophysical and pharmacological approaches to investigate this role using single- and higher-order *Arabidopsis* (*Arabidopsis thaliana*) *cp26* mutants, examining its relationship to known NPQ regulators (Photosystem II subunit S, PsbS, violaxanthin de-epoxidase, and the pH gradient across the thylakoid membrane). *cp26* mutants showed significantly reduced maximum PSII quantum efficiencies (F_v_/F_m_) in darkness, indicating a constitutively quenched state, further confirmed by fluorescence lifetime measurements. Destabilized PSII-LHCII supercomplexes observed in native gel electrophoresis and tighter PSII supercomplex packing were potential causes, with no other antenna proteins capable of rescuing this phenotype. In addition, the *cp26* mutants exhibited altered NPQ capacity—modest in single mutants but substantial in double mutants—independent of PsbS and violaxanthin de-epoxidase. Together, these results show that CP26 is not involved in qE or qZ but may primarily play an indirect role in apparent NPQ responses via PSII-LHCII supercomplex organization.

## Teaser

Experiments with higher-order *cp26* mutants demonstrate that CP26 is not involved in qE or qZ but may affect apparent NPQ responses via its role in PSII-LHCII supercomplex organization.

## Introduction

Under sub-optimal environmental conditions, such as high light and/or cold stress, photoprotective mechanisms protect the photosynthetic electron transport chain from photooxidative damage by preventing the formation of harmful reactive oxygen species (Bassi and Dall’Osto 2021). The dissipation of excess light energy as heat is commonly referred to as non-photochemical quenching (NPQ) (for recent reviews, see Ruban and Wilson 2021; Van Amerongen and Croce 2025). The fastest NPQ component is termed energy-dependent quenching (qE) and, in plants, relies on the Photosystem II (PSII) subunit S (PsbS) as a lumenal pH sensor (Li et al. 2000, 2002a; Nicol and Croce 2021). Upon light exposure, lumen-exposed glutamate residues in PsbS are protonated (Liguori et al. 2019; Li et al. 2002b, 2004), inducing conformational changes and monomerization of PsbS dimers (Bergantino et al. 2003; Krishnan et al. 2017). The main qE quenching site Q1 has been hypothesized to be located in detached LHCII aggregates (Horton et al. 1991; Mullineaux et al. 1993; Miloslavina et al. 2008, 2011; Goral et al. 2012; Tian et al. 2015; Natali et al. 2016; Adams et al. 2018; Shukla et al. 2020; Tutkus et al. 2021; Wilson et al. 2022); however, the actual role of PsbS in forming this qE site remains to be determined (Marulanda Valencia and Pandit 2024). PsbS monomers could intercalate into the light-harvesting complexes around PSII (LHCII antenna system) and facilitate detachment of loosely (L) and moderately (M) bound LHCII trimers from the PSII-LHCII supercomplexes, thereby rearranging the major antennae into detached LHCII aggregates (Kiss et al. 2008; Betterle et al. 2009; Wilk et al. 2013; Ware et al. 2015; Correa-Galvis et al. 2016; Sacharz et al. 2017; Daskalakis et al. 2019; Pawlak et al. 2020; Nicol and Croce 2021). In addition, recent work by Chen et al. (2025) suggests that the dimer-to-monomer transition in itself is a prerequisite for qE induction, perhaps by promoting lateral mobility of PsbS from the grana core to the margins. In parallel to the low pH-triggered changes in PsbS, the reversible xanthophyll cycle is activated through protonation of the enzyme violaxanthin de-epoxidase (VDE), which converts violaxanthin into antheraxanthin and zeaxanthin upon high light exposure, thereby enhancing qE. Aside from the putative qE quenching site in aggregated trimeric LHCII, zeaxanthin and the xanthophyll lutein bound to the minor antennae appear to form transient radical cations, which could potentially be part of an independent NPQ mechanism (Holt et al. 2005; Ahn et al. 2008; Avenson et al. 2008; Li et al. 2009; Pinnola et al. 2016; Leuenberger et al. 2017).

Another quenching site (Q2) has been proposed in the strongly bound S-LHCII trimers attached to PSII and possibly in the minor antennae (CP29, CP26, and CP24), which are located between the major LHCII trimers and the PSII core. NPQ occurring in the Q2 site could account for the slower phase of zeaxanthin-dependent NPQ induction (qZ, several minutes to tens of minutes timescale), relying solely on the activity of the xanthophyll cycle (Miloslavina et al. 2008; Nilkens et al. 2010), although this mechanism has been debated (Saccon et al. 2020). *In vitro* reconstitution of recombinant LHCII proteins with the xanthophyll pigments revealed that CP26 binds zeaxanthin much more efficiently than any other LHCII protein (Morosinotto et al. 2002). Moreover, CP26 is the only minor antenna protein that has been shown to undergo a conformational change upon binding zeaxanthin—independently from the presence of PsbS—resulting in a shift in isoelectric point (pI) and reduced fluorescence levels (Dall’Osto et al. 2005). *In vitro* chlorophyll-binding site analyses and *Chlamydomonas* mutant analysis also suggested that CP26 could be involved in quenching (Ballottari et al. 2009, 2013; Cazzaniga et al. 2020, 2023).

To follow up these findings, we investigated the contribution of the minor antenna protein CP26 to NPQ and its relationship to different qE and qZ components *in vivo* by studying the single knockout mutant *cp26*, as well as double mutants with modified PsbS (*cp26 npq4* and *cp26 L17*) and VDE (*cp26 npq1*) levels. We found that single and higher-order *cp26* mutants had destabilized PSII-LHCII supercomplexes and maintained a quenched state after dark acclimation. Under high light, however, *cp26* mutants displayed altered NPQ levels compared to their controls, particularly during the later phase of NPQ induction. Consistent with previous *in vitro* analyses (Dall’Osto et al. 2005), altered NPQ in *cp26* mutants was independent of PsbS and VDE. However, the effect was removed by inhibition of lumen acidification or blocking of protonatable residues. Altogether, these results show that CP26 does not contribute significantly to qE or qZ, but its absence leads to a quenched dark-acclimated state and altered slow phase NPQ dynamics, likely due to PSII-LHCII supercomplex reorganization.

## Results

### *Arabidopsis cp26* mutants exhibit lower PSII efficiencies in darkness due to a pre-quenched state

To investigate the relationship between CP26 and the key proteins for initiating qE and qZ, PsbS, and VDE, the *Arabidopsis* (*Arabidopsis thaliana*) *cp26* T-DNA insertion line SALK-014869C was crossed with the PsbS knockout line *npq4*, the PsbS overexpression line L17, and the VDE knockout line *npq1* (for molecular confirmation of double mutants, see Figure S1).

Using biophysical and biochemical assays, the *cp26* single and double mutants were assessed for their photosynthetic and photoprotective capacities in comparison to their respective wild-type (WT)/single mutant controls.

**Figure 1:**
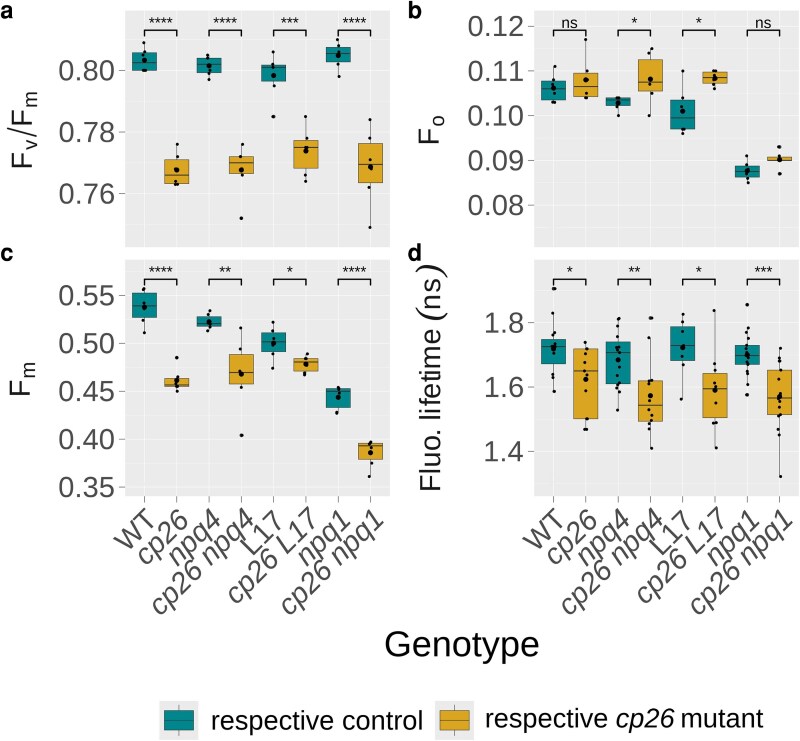
Dark-acclimated chlorophyll fluorescence parameters of *cp26* mutants. (a) Maximum quantum yield of Photosystem II, F_v_/F_m_, calculated from minimum (b F_o_) and maximum (c F_m_) chlorophyll fluorescence levels. (d) Chlorophyll fluorescence lifetime values (in nanoseconds). Measurements were done on intact leaves by applying a saturating pulse following dark acclimation. Data were collected from six biological replicates (n = 6) for pulse-amplitude-modulated chlorophyll fluorescence measurements and 7 to 17 biological replicates (n = 7 to 17) for chlorophyll fluorescence lifetime measurements. Boxplots contain individual data points (black dots), the median (black line inside the box), and the mean (larger black dot inside the box). Boxes show upper and lower quartiles, whiskers show the 1.5x interquartile range, points outside whiskers show outliers. Significant differences between control genotypes (wild-type WT, *npq4*, L17, and *npq1*; green boxes) and *cp26* mutants (*cp26*, *cp26 npq4*, *cp26 L17,* and *cp26 npq1*; yellow boxes) are indicated by asterisks (Student's *t*-test). Significance levels: ns = no significance, * = *P* < 0.05, ** = *P* < 0.01, *** = *P* < 0.001, and **** = *P* < 0.0001.

The maximum quantum efficiency of PSII (F_v_/F_m_) was determined after 75 min of dark acclimation by measuring pulse-amplitude-modulated (PAM) chlorophyll fluorescence from intact leaves. All *cp26* mutants showed significantly lower F_v_/F_m_ values compared to their controls (0.77 vs. 0.80, *P* < 0.001; Fig. 1a). A decrease in F_v_/F_m_ either suggests an increase in the minimum fluorescence (F_o_) or a lower dark-acclimated maximum fluorescence (F_m_). F_o_ levels of *cp26* mutants were slightly elevated (significantly so for *cp26 npq4* vs. *npq4* and *cp26 L17* vs. *L17*; *P* < 0.05; Fig. 1b). However, the decrease in F_v_/F_m_ predominantly originated from the F_m_ values, which were significantly lower in all *cp26* single and double mutants compared to their respective controls (0.001 < *P* < 0.05, Fig. 1c), indicating a quenched state even in the absence of light. Time-correlated single photon counting (TCSPC) measurements on intact leaves further corroborated these data by revealing a decrease in chlorophyll fluorescence lifetime of closed PSII centers by approximately 0.1 ns in the absence of CP26 across all genotypic pairs (Fig. 1d). Note that this quenched state does not imply a regulatory mechanism, but is more likely to derive from structural perturbations of PSII-LHCII supercomplexes.

Following F_v_/F_m_ measurements, chlorophyll fluorescence was recorded during two consecutive cycles of high light (1,000 µmol photons m^−2^ s^−1^ for 20 min) and dark (10 min). PSII efficiencies during light phases (F_q_′/F_m_′) were similar between all genotypes, with small differences only observed in the comparison between L17 and the *cp26 L17* double mutant (Figure S2b, bottom panel). In contrast, during the intermittent dark phases, most CP26-deficient plants had significantly lower F_v_′/F_m_′ values (PSII efficiencies during dark phases; equivalent to F_q_′/F_m_′ in the light) than their respective controls (Figure S2). Additionally, PSII quantum efficiencies were significantly decreased in the second dark phase compared to the first. Consistent with the lack of differences during illumination, no differences in electron transfer rate in PSII, (ETR(II); Figure S3a&b)) were detected. Net CO_2_ assimilation rate (A_net_) was also similar in steady state (Figure S3d) and was only slightly lower in *cp26* than WT in the first minute during the second high light induction (Figure S3c).

### Absence of CP26 affects NPQ during illumination

**Figure 2:**
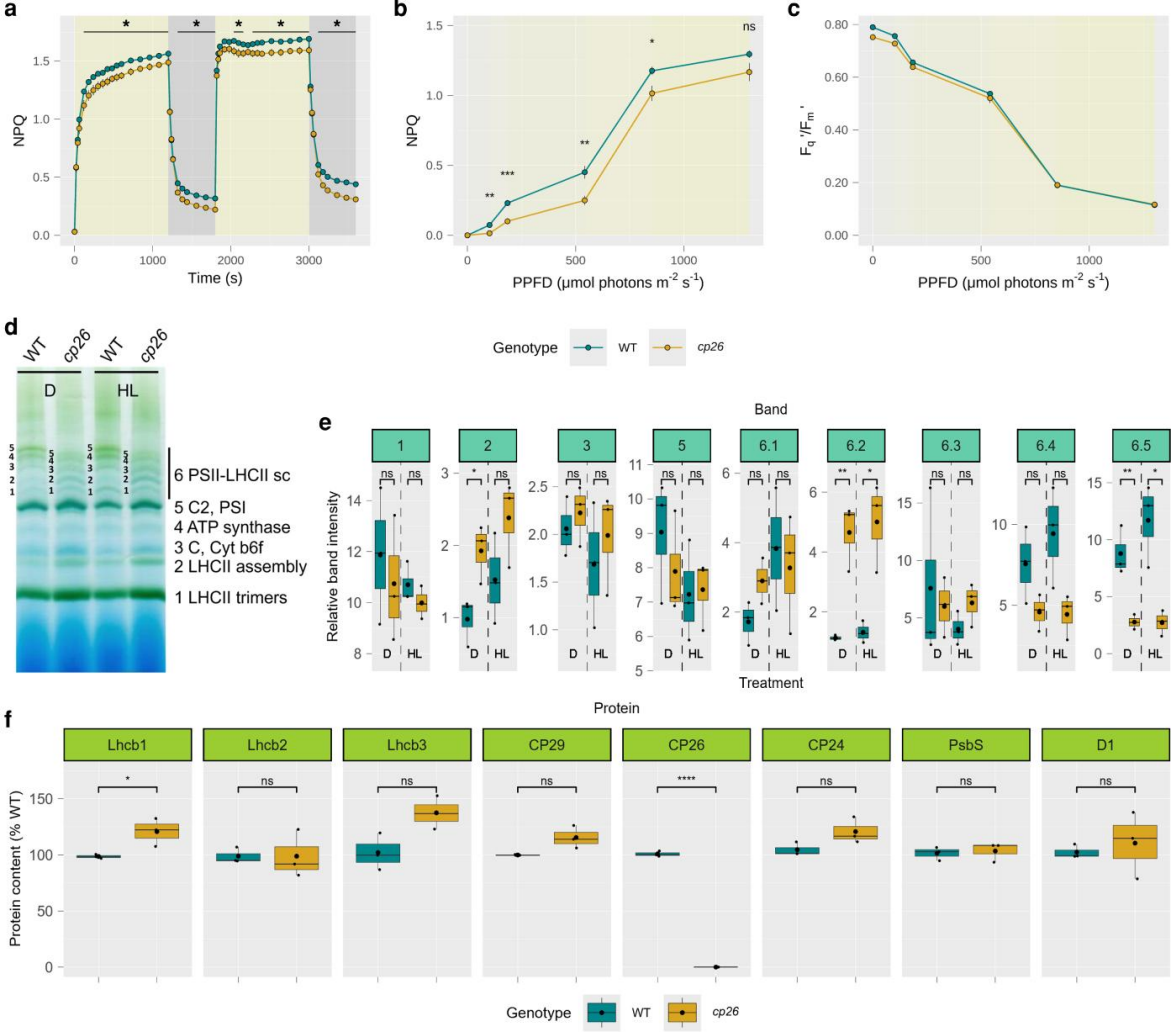
Non-photochemical quenching (NPQ), photosystem II (PSII) quantum yield, and PSII antenna composition in *cp26*. (a) NPQ during high light (1,000 µmol photons m^−2^ s^−1^ for 20 min; yellow background) and dark phases (10 min; dark gray background) in two consecutive cycles. (b–c) Light response curves from plants exposed to increasing light intensities (photosynthetic photon flux density, PPFD). PSII parameters (b NPQ and c PSII efficiency, F_q_′/F_m_′) were calculated by applying a saturating pulse at the end of each light phase. (d–f) Protein complex analyses of thylakoid extracts. (d) Blue Native-Polyacryalamid Gel Electrophoresis gel from dark (D) and high light (HL)-treated samples, representative image from three biological replicates (n = 3). Labeled bands correspond to: 1) Light-harvesting complex II (LHCII) trimers, 2) LHCII assembly, 3) PSII monomers (C) and Cytochrome b6f, 4) adenosine triphosphate (ATP) synthase, 5) PSII dimers and PSI, 6) PSII-LHCII supercomplexes, according to Järvi et al. (2011). (e) Band intensities corresponding to the bands in (d) determined with ImageJ and normalized to the intensity of band 4. (f) Western blot analyses with ImageJ from three biological replicates (n = 3) of high light-treated plants to quantify the abundance of thylakoid membrane proteins relative to the wild-type (WT). Data in (a)–(c) were collected from six biological replicates (n = 6) for pulse-amplitude-modulated fluorescence measurements. Line plots show mean values with error bars indicating the standard error of the mean. The asterisk designates significant differences between WT (green circles) and *cp26* (yellow circles) (two-way repeated measures analysis of variance with following non-paired *t*-test as post-hoc test). In (c), the Genotype:PPFD interaction effect was not significant (*P* = 0.084). Boxplots (n = 3) contain individual data points (black dots), the median (black line inside the box) and the mean (larger black dot inside the box). Boxes show upper and lower quartiles, whiskers show the data range. Significant differences between WT (green boxes/circles) and *cp26* mutant (yellow boxes/circles) are indicated by asterisks (Student's *t*-test). Significance levels: ns = no significance, * = *P* < 0.05, ** = *P* < 0.01, *** = *P* < 0.001 and **** = *P* < 0.0001.

NPQ was calculated from the same two cycles of 20 min high light and 10 min darkness (Fig. 2a). During high light exposure, NPQ in WT was rapidly induced during the first 2 min until it reached a substantially slower phase of induction, with a maximum NPQ value of ∼1.6 at the end of the first high light phase. Similarly, during the first transition to darkness, NPQ initially decreased rapidly, followed by a slower phase of relaxation. In the second cycle of high light, NPQ reached a plateau more quickly at a higher level (∼1.7) compared to the last data point of the preceding high light cycle, which could be explained by the accumulation of zeaxanthin during the previous high light phase. The *cp26* plants displayed the same trends but had slightly, although significantly, lower NPQ levels than WT plants in the slower phases of NPQ induction (∼95% of WT) and relaxation (∼70% of WT).

To explore the light-intensity dependence of these *cp26*-associated differences, NPQ and photosynthetic efficiency were also measured across five different light intensities spanning the limiting to saturating range (Fig. 2b, c). NPQ in *cp26* was generally lower than in the WT, although the difference in NPQ between both genotypes decreased at higher light intensities (Fig. 2b). F_q_′/F_m_′ was slightly lower in *cp26* at low light, but the difference decreased with higher light intensities, and both genotypes were similar at light intensities above 200 µmol photons m^−2^ s^−1^ (Fig. 2c).

CP26 is a subunit of the light-harvesting complex located at the outer interface of strongly bound S-LHCII trimers and the PSII core, that is, in the corner of the C2S2M2 PSII supercomplex. Loss of CP26 has previously been associated with less stable PSII-LHCII supercomplexes, and some papers have reported substitution by other minor antenna proteins, CP29 and CP24 (Andersson et al. 2001; De Bianchi et al. 2008; Miloslavina et al. 2011; Goral et al. 2012). To verify these previous observations, thylakoid membrane protein complexes were isolated from dark-acclimated and high light-treated plants, separated in their native state with blue native-polyacrylamide gel electrophoresis (BN-PAGE), and the contents of LHCII proteins were determined from high light-treated samples by denaturing western blot analyses (Fig. 2d–f). The bands on the BN-PAGE gel were annotated with thylakoid protein complexes according to Järvi et al. (2011). The largest PSII-LHCII cluster (the top WT band 6.5) was significantly less abundant (and shifted to a slightly smaller size) in *cp26*, while the smaller supercomplexes in band 6.2 instead showed an increased abundance in *cp26*, as did band 2 “LHCII assembly,” corresponding to free M-LHCII trimers and the heterodimer CP29/CP24 (Aro et al. 2005; Sárvári et al. 2022), more so in dark samples than high light samples. Analyses of the relative contents of LHCII proteins in the thylakoid membrane fraction revealed an increase in Lhcb1 (20.8%; *P* = 0.039), Lhcb3 (37.5%; *P* = 0.051), and CP29 (15.5%; *P* = 0.057) protein levels in *cp26* compared to WT (Fig. 2f), although the latter two were not statistically significant. It should be noted that since protein samples were loaded based on chlorophyll content and CP26 contributes about 9% to the total chlorophyll content in a C2S2M2 PSII-LHCII supercomplex, this could result in a slight overloading of other proteins in the *cp26* samples. If this effect were significant, it should apply equally to all remaining thylakoid proteins. However, no increases were observed for D1 protein, which was used as a loading control and Coomassie staining also did not show any evidence of unequal loading (see Fig 2f and Figure S4 and the Supplementary File S1).

### *cp26* mutants in backgrounds with contrasting PsbS levels had lower NPQ than their controls in the slower phase of NPQ induction/relaxation

**Figure 3:**
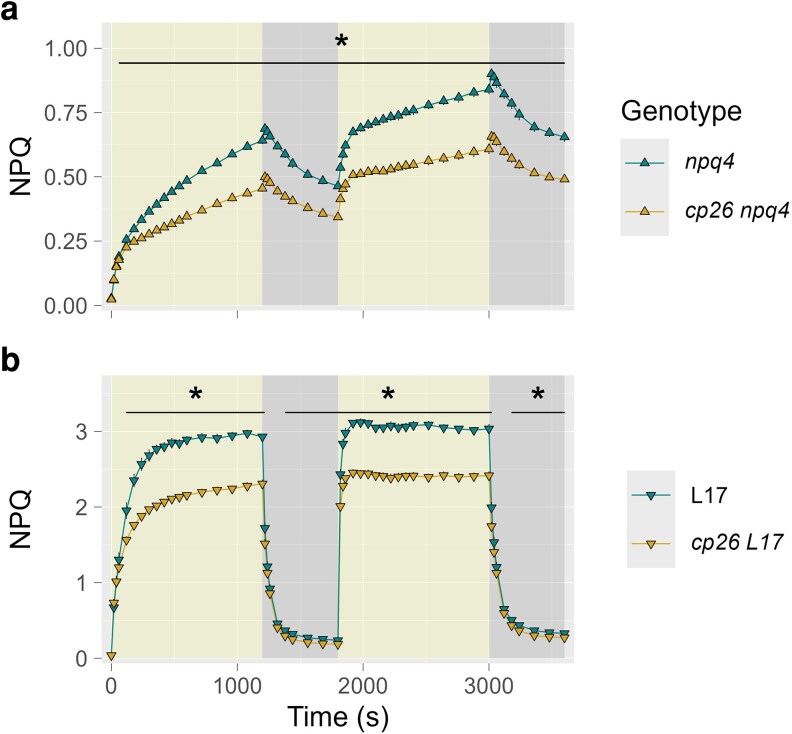
Fluorescence parameters of *cp26* mutants crossed with contrasting photosystem II subunit S (PsbS) alleles (knockout = *npq4* and overexpression = L17). Non-photochemical quenching (NPQ) measurements of *cp26* double mutants either lacking PsbS (*cp26 npq4*, yellow upward-pointing triangles in (a) or overexpressing PsbS (*cp26 L17*, yellow downward-pointing triangles in (b) and their respective controls *npq4* and L17 (green triangles) during high light induction (1,000 µmol photons m^−2^ s^−1^ for 20 min) and dark relaxation (10 min) in two consecutive cycles. Line plots show mean values from six biological replicates (n = 6), with error bars indicating the standard error of the mean. The asterisk represents significant differences between two genotypes at each measurement point (two-way repeated measures analysis of variance with following non-paired *t*-test as post-hoc test). Significance level: * = *P* < 0.05.

A potential interaction between CP26 and PsbS was evaluated *in vivo* by crossing the *cp26* mutant with the PsbS knockout line *npq4* (*cp26 npq4*) and the PsbS overexpression line L17 (*cp26 L17*). This allowed comparisons of chlorophyll fluorescence parameters between lines with varying levels of PsbS protein (no PsbS, WT PsbS level, and PsbS overexpression) in the presence and absence of CP26, using the same high light/dark illumination sequence described above (Fig. 3).

Consistent with the prominent role of PsbS as a regulator of energy-dependent qE, *npq4* mutants exhibited approximately 50% less NPQ than WT during high light induction with minimal relaxation of the established NPQ in the dark (Fig. 3a vs. Figure 2a). L17 mutants had 2-fold higher NPQ amplitudes than WT during high light but reached lower NPQ than WT upon relaxation in the dark phase (Fig. 3b vs. Figure 2a) with NPQ levels of 0.32 vs. 0.24 in dark phase 1 and 0.44 vs. 0.33 in dark phase 2 for WT and L17, respectively (*P* < 0.005). In both cases, the double mutants *cp26 npq4* and *cp26 L17* displayed significantly lower NPQ than their controls during the slower phase of NPQ induction (after 60 s), which remained lower during the first dark phase and the following high light/dark cycle in *cp26 npq4*. In PsbS overexpression lines, NPQ levels showed a pronounced difference at the end of the high light phases (L17: NPQ = 3; *cp26 L17*: NPQ = ∼2.4), while after 1 min of both dark phases, this difference was no longer apparent (at time point 60 s in the dark phases, both genotypes had NPQ levels of ∼1), indicating slower qE relaxation in *cp26 L17*. Nevertheless, by the end of the dark phases, *cp26 L17* reached lower NPQ values than L17.

Protein complex stoichiometries in the double mutants of *cp26* with *npq4* and *L17* (Figure S4) showed similar trends to the analysis of the single *cp26* mutant (Fig. 2d–f). While most double *cp26* mutants seemed to have a higher abundance of detached LHCII assemblies relative to their controls (band 2; Figure S4a, b), the largest PSII-LHCII supercomplexes visible in *npq4* and L17 were consistently absent or shifted upon loss of CP26 (Figure S4a). Densitometric analyses of western blots against LHCII protein subunits of these samples revealed an increase in Lhcb3 (28.4%; *P* = 0.092) and CP24 (16.7%; *P* = 0.018) protein abundance in *cp26 L17* compared to L17 (Figure S4c), although protein levels varied significantly between the three replicates. Interestingly, the different abundances of PsbS between *npq4*, WT, and *L17* did not seem to affect the stability of different PSII-LHCII supercomplexes.

### The difference in NPQ due to CP26 deficiency persists when the xanthophyll cycle is blocked

**Figure 4:**
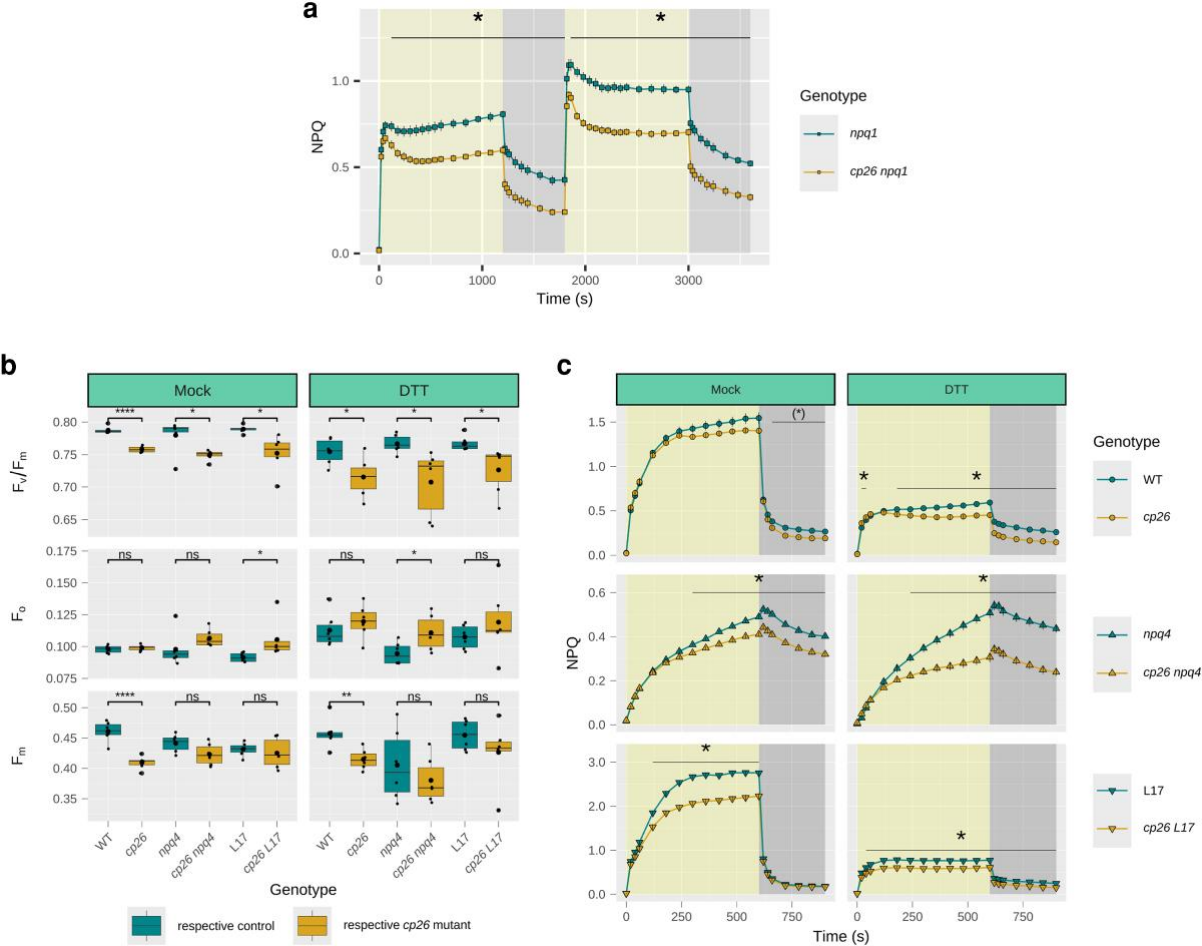
Non-photochemical quenching (NPQ) in mutants lacking the xanthophyll cycle enzyme Violaxanthin de-epoxidase (VDE, *npq1*), and photosystem II (PSII) quantum yields and NPQ in mutants with contrasting PsbS alleles (knockout = *npq4* and overexpression = L17) infiltrated with dithiothreitol (DTT). (a) NPQ of the *cp26* double mutant *cp26 npq1* (yellow squares) compared to the single mutant *npq1* (green squares) during two consecutive high light/dark cycles (20/10/20/10 min). (b–c) Leaf infiltration assays of wild-type (WT, green circles), *npq4* (green upward-facing triangles), and L17 (green downward-facing triangles) and respective *cp26* mutants (yellow symbols) using a HEPES/KOH (pH 7.0) buffer as a control (Mock) and the VDE inhibitor DTT. (b) Maximum quantum yield of PSII, F_v_/F_m_, calculated from minimum (F_o_) and maximum (F_m_) chlorophyll fluorescence levels. (c) NPQ traces were recorded during one high light (1,000 µmol photons m^−2^ s^−1^ for 10 min, yellow background) and one dark cycle (5 min, dark-gray background) by applying saturating pulses. Line graphs show mean values from six biological replicates (n = 6), with error bars indicating the standard error of the mean. The asterisk represents significant differences between the two genotypes at each measurement point (two-way repeated measures analysis of variance with following non-paired *t*-test as post-hoc test). Boxplots contain individual data points (black dots), the median (black line inside the box) and the mean (larger black dot inside the box). Boxes show upper and lower quartiles, whiskers show the 1.5× interquartile range, points outside whiskers show outliers. Significant differences between control genotypes (WT, *npq4,* and L17; green boxes) and *cp26* mutants (*cp26*, *cp26 npq4,* and *cp26 L17*; yellow boxes) are indicated by asterisks (Student's *t*-test). Significance levels: ns = no significance, * = *P* < 0.05, ** = *P* < 0.01, *** = *P* < 0.001, and **** = *P* < 0.0001.

Genetic and pharmacological approaches were used to assess the contribution of the xanthophyll cycle to NPQ in *cp26* (Fig. 4). First, the *cp26* mutant was crossed with the VDE knockout mutant *npq1*, and NPQ was recorded during two cycles of high light/dark phases as described above (Fig. 4a). The *npq1* mutant lacks violaxanthin de-epoxidase (VDE) activity and is therefore unable to form zeaxanthin via the reversible xanthophyll cycle. Since zeaxanthin contributes to both qE and qZ components, NPQ levels in this mutant were 50% lower compared to the WT (Fig. 4a vs. Figure 2a) and showed a small decline in the slower phase of WT NPQ induction. Throughout the high light measurement period, NPQ was significantly lower in the *cp26 npq1* double mutant compared to *npq1,* except during the fast-inducing phases (20 to 60 s), with a more pronounced initial decline in NPQ beyond 1 min in each of the two high light phases (Fig. 4a).

Additionally, leaf segments of *cp26*, *cp26 npq4,* and *cp26 L17* were infiltrated with dithiothreitol (DTT) diluted in 20 mM HEPES/KOH buffer (pH 7.0) to inhibit VDE activity, and chlorophyll fluorescence was recorded during a shorter 10/5 min high light/dark cycle (Fig. 4b/c). Consistent with the *cp26 npq1* findings, DTT infiltration did not affect the overall trends of the dark values F_v_/F_m_, F_o,_ and F_m_ (Fig. 4b, *P* = 0.634, 0.215, and 0.065, respectively, two-way analysis of variance (ANOVA) for Genotype:treatment interaction) and lower NPQ induction, associated with the lack of CP26, persisted in both single and double mutants (Fig. 4c). This is despite the fact that absolute NPQ levels decreased by two-thirds in DTT-infiltrated samples of WT, *cp26*, *L17,* and *cp26 L17* due to the inhibitory effect of blocking zeaxanthin on qE formation. These observations clearly showed that the effect of CP26 knockout on NPQ induction persists when the xanthophyll cycle is impaired.

### Inhibition of the proton gradient or blocking of protonatable residues abolished the NPQ induction difference between *cp26* and WT

**Figure 5:**
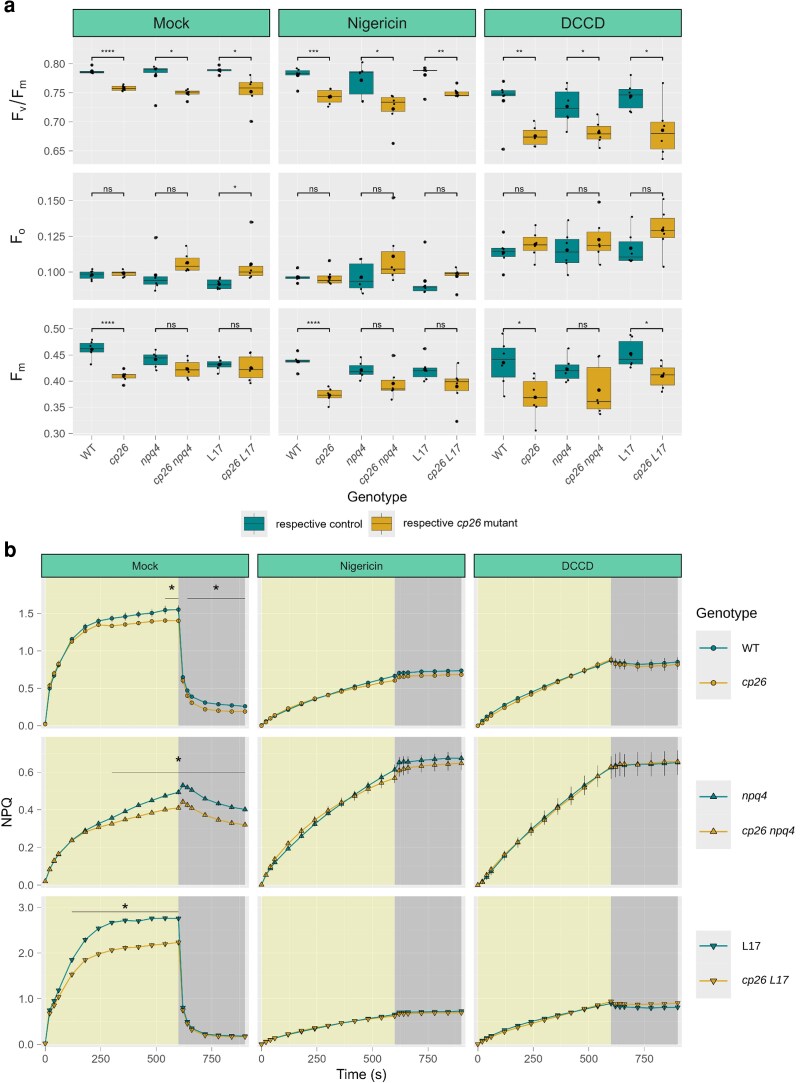
Dark-acclimated chlorophyll fluorescence parameters and non-photochemical quenching (NPQ) induction in leaves of *cp26* and double mutants with contrasting photosystem II subunit S (PsbS) alleles (knockout = *npq4* and overexpression = L17) infiltrated with nigericin or N,N′-dicyclohexylcarbodiimide (DCCD). Leaves were infiltrated with HEPES/KOH (pH 7.0) buffer as a control (Mock, replotted from Fig. 4b/c), nigericin, an inhibitor of the proton gradient across the thylakoid membrane, and DCCD, an inhibitor of protonatable protein residues in the thylakoid lumen. (a) Maximum quantum yield of PSII, F_v_/F_m_, calculated from minimum (F_o_) and maximum (F_m_) chlorophyll fluorescence levels. (b) NPQ was recorded for the genotype pairs: wild-type (WT) vs. *cp26* (green and yellow circles, respectively); *npq4* vs. *cp26 npq4* (green and yellow upward-pointing triangles, respectively); and L17 vs. *cp26 L17* (green and yellow downward-pointing triangles, respectively) during one high light (1,000 µmol photons m^−2^ s^−1^ for 10 min, yellow background) and dark cycle (5 min, dark-gray background). Boxplots contain individual data points (black dots), the median (black line inside the box) and the mean (larger black dot inside the box) from six biological replicates (n = 6). Boxes show upper and lower quartiles, whiskers show the 1.5× interquartile range, points outside whiskers show outliers. Significant differences between control genotypes (WT, *npq4,* and L17; green boxes) and *cp26* mutants (*cp26*, *cp26 npq4,* and *cp26 L17*; yellow boxes) are indicated by asterisks (Student's *t*-test). Line graphs show mean values from six biological replicates (n = 6) with error bars indicating the standard error of the mean. The asterisk represents significant differences between the two lines at each measurement point (two-way repeated measures analysis of variance with following non-paired *t*-test as post-hoc test). In (b), Genotype:time interaction effects were not significant for nigericin and DCCD treatments (*P* > 0.05). Significance levels: ns = no significance, * = *P* < 0.05, ** = *P* < 0.01, *** = *P* < 0.001, and **** = *P* < 0.0001.

The above results show that the effect of CP26 knockout on NPQ, induced upon illumination, is independent of the pH sensor protein PsbS and the xanthophyll cycle. To further investigate the impact of lumen pH on NPQ in *cp26*, the effects of the inhibitors nigericin (Gilmore et al. 1995) and *N,N*′-dicyclohexylcarbodiimide (DCCD) (Ruban et al. 1992; Li et al. 2004) on NPQ in the *cp26* mutants with varying PsbS levels were tested (Fig. 5). Nigericin collapses the proton gradient across the thylakoid membrane, while DCCD binds protonatable protein residues under acidic pH. Measurements of F_v_/F_m_, F_o,_ and F_m_ showed that PSII parameters were more strongly affected by DCCD infiltration than by nigericin infiltration. Nevertheless, the relative changes in F_v_/F_m_, F_o,_ and F_m_ due to loss of CP26 trended similarly to the mock infiltration despite the lack of ΔpH (Fig. 5a), and there was no Genotype:treatment interaction effect for any of the three dark-adapted parameters (*P* = 0.686, 0.772, and 0.298, respectively; two-way ANOVA).

Collapsing the pH gradient across the thylakoid lumen using nigericin or blocking protonatable residues using DCCD should abolish all pH-dependent NPQ. Indeed, NPQ traces of all genotypes treated with nigericin and DCCD showed monotonous increases in NPQ during illumination (Fig. 5b), reaching NPQ levels of ∼0.7 at the end of the high light phase and showing negligible NPQ relaxation in the dark. In the absence of PsbS (*npq4* mutants), the difference in NPQ between *npq4* and *cp26 npq4* in the mock treatment persists (similar to Fig. 3a). Interestingly, nigericin and DCCD infiltrations abolished these *cp26-*associated differences in NPQ during illumination, despite the continued impact of CP26 knockout on dark-acclimated fluorescence parameters. This suggests that the decrease in NPQ induction observed in *cp26* mutants does not originate entirely from a partially pre-quenched F_m_ in dark-acclimated samples but that slow-phase dynamics of NPQ during acidification of the thylakoid lumen are also affected, independent of PsbS.

### Proton motive force build-up and partitioning are not affected in *cp26* mutants

**Figure 6:**
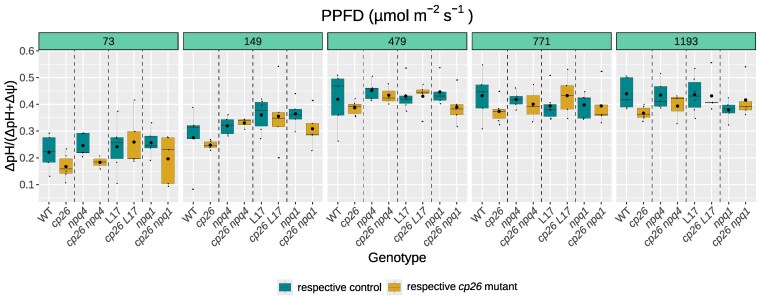
Evaluation of the trans-thylakoid proton gradient ΔpH in single and double *cp26* mutants across a range of increasing light intensities. Electrochromic shift signal (ECS) was measured at 515 nm during light-to-dark transitions in a light response curve (5 min light followed by 5 min dark at the indicated photosynthetic photon flux density, PPFD) to determine the proton motive force (pmf) and its components ΔpH and ΔΨ according to Cruz et al. (2001). The proportion of ΔpH to pmf (ΔpH + ΔΨ) was calculated from three to six biological replicates (n = 3 to 6). Boxplots contain individual data points (black dots), the median (black line inside the box) and the mean (larger black dot inside the box). Boxes show upper and lower quartiles, whiskers show the 1.5x interquartile range, points outside whiskers show outliers. Significant differences between *cp26* mutants (*cp26*, *cp26 npq4*, *cp26 L17,* and *cp26 npq1*; yellow boxes) and their respective controls (wild-type WT, *npq4*, L17, and *npq1*; green boxes) were determined using two-way repeated measures analysis of variance (*P* > 0.05 for all Genotype:PPFD interaction effects, that is, no significant differences were observed).

To find out if differences in NPQ induction between single and *cp26* double mutants arose due to an effect of CP26 deletion on the formation of trans-membrane ΔpH, the proton motive force (pmf) was assessed *in vivo* via electrochromic shift (ECS) measurements (Figure S5). However, no impact on the proton conductivity of the ATP synthase, gH^+^ (Figure S5a), the steady-state proton flux rate, vH^+^ (Figure S5b), or overall pmf (Figure S5c) was observed for the single *cp26* mutant or the three double mutants (*cp26 npq4*, *cp26 L17,* and *cp26 npq1*). Similarly, partitioning of pmf into the proton gradient and electrostatic potential across a range of light intensities was not affected by CP26 mutation in either single or double mutants (Fig. 6).

### Accounting for differences in F_m_ does not remove the effects of CP26 knockout on the slower phase of NPQ induction

NPQ calculations based on PAM fluorescence follow the Stern-Volmer equation, NPQ = (F_m_/F_m_′) – 1, and are therefore dependent on the maximum chlorophyll fluorescence value F_m_, which is initially measured after dark acclimation. As discussed above, F_m_ is significantly lower in the *cp26* mutant, introducing a confounding factor for NPQ quantification. To determine whether the F_m_ reduction in *cp26* mutants underpinned the observed differences in NPQ during the slow phase of induction, we calculated NPQ(T) based on a theoretical model that assumes a standard F_v_/F_m_ value of 0.83 (Tietz et al. 2017). Interestingly, NPQ(T) values were significantly shifted to higher levels than their controls in all single and double *cp26* mutants (Figure S6a, b, and d) except for *cp26 L17*, which showed lower NPQ(T) values than *L17* (Figure S6c). In each case, the assumption of equal F_m_ in the NPQ(T) calculation did not remove the effect of CP26 deletion on NPQ induction, as significant differences between the genotype pairs remained. Nevertheless, the fact that the effect of CP26 knockout on NPQ(T) changes directionality relative to pairwise differences in NPQ indicates that the overall decrease in NPQ induction in *cp26* in response to light is likely somewhat dependent on the pre-quenched F_m_.

## Discussion

Of the minor LHCII antennae, CP29 has been the main target of NPQ research (Feng et al. 2013; Betterle et al. 2015, 2017; Gacek et al. 2020; Guardini et al. 2020, 2022; Mascoli et al. 2020; Cignoni et al. 2021; Sardar et al. 2022, 2024; Elias et al. 2023) as it directly connects major LHCII trimers to the PSII core, and its deletion showed an initial impact on NPQ induction upon high light exposure (De Bianchi et al. 2011; Miloslavina et al. 2011; Guardini et al. 2020). In addition, the CP24 protein is structurally dependent on the presence of CP29, and a *cp24* knockout mutant was also shown to be impaired in overall NPQ capacity (Kovács et al. 2006; De Bianchi et al. 2008; Chen et al. 2018). On the other hand, the deletion of CP26 was found to have only a minor influence on NPQ in land plants (De Bianchi et al. 2008), which was confirmed by our results. Previous *in vitro* studies of CP26 suggested a unique connection to and dependency on the xanthophyll zeaxanthin (Morosinotto et al. 2002; Dall’Osto et al. 2005). Although our results show both a dark quenched state and a perturbation of NPQ induction, which was modest in *cp26* single and substantial in double mutants, neither effect relies on zeaxanthin formation. In the following sections, we discuss these phenomena in more detail. To facilitate easy comparison with previous studies, our experimental plants were grown under a single set of growth conditions. However, the environmental conditions during growth, such as intensity and spectral quality of the light regime, can also affect NPQ responses (e.g. Mateo et al. 2006; Górecka et al. 2020). In addition, structural rearrangement of PSII in field-grown plants compared to those from controlled conditions has been suggested (Wituszyńska et al. 2013a, 2013b). We are therefore not able to rule out that different, less benign growth conditions could affect some of our reported observations here, as well as those in previous work.

### The pre-quenched dark state in *cp26* mutants is likely to be a sustained form of photoinhibitory quenching

Analysis of the maximum PSII quantum efficiency after dark acclimation revealed that *cp26* mutants had lower F_v_/F_m_ values compared to their controls (Fig. 1a), primarily due to decreases in F_m_ (Fig. 1c), as well as a slight increase in F_o_ (Fig. 1b). These observations are consistent with previous observations for mutants lacking CP26 (De Bianchi et al. 2008, 2011; Chen et al. 2018) and likely indicate a structural impairment leading to photoinhibitory quenching. This is in line with recent work, where PSII supercomplexes in the absence of CP26 showed slightly altered conformation and were found to be packed more tightly in semicrystalline arrays, with non-parallel associations entirely lacking (Vánská et al. 2025). If the pre-quenched state in darkness is indeed photoinhibitory quenching, it should be independent of the prerequisites of initiating qE and qZ, that is, lumen acidification and zeaxanthin formation. Indeed, our results confirm that when these were inhibited either genetically in the double mutants *cp26 npq4* and *cp26 npq1* or via leaf infiltration with DTT, nigericin, or DCCD, the dark-quenched state in *cp26* persisted (Figs. 1a, Fig. 4b, and Fig. 5a), although only differences in F_v_/F_m_ were consistently significant. In addition, the observation that NPQ traces in *cp26* single and double mutants closely resembled controls in nigericin or DCCD infiltrated leaves demonstrates that the pre-quenched state is not photoprotective.

Under dark conditions, the PSII core and LHCII proteins form large supercomplexes to maximize light interception when photons are limited. CP26 is not essential for supercomplex formation (Yakushevska et al. 2003; Van Oort et al. 2010) but might stabilize and fine-tune its structure in the thylakoid membrane. BN-PAGE analysis of *cp26* thylakoid extracts (Fig. 2d/e and Figure S4a/b) revealed a decrease in the abundance of the highest order PSII-LHCII supercomplexes and a trend toward increased free LHCII assemblies (M-LHCII/CP29/CP24; Aro et al. 2005; De Bianchi et al. 2011; Sárvári et al. 2022). Notably, these effects are likely exacerbated by detergent solubilization, which disrupts weak protein interactions. Nevertheless, these results are consistent with prior findings of less stable supercomplexes in CP26 antisense lines (Yakushevska et al. 2003) due to the loss of CP26. Image analysis of ordered PSII arrays also showed that adjacent supercomplexes are interspersed by CP26 in WT, but this structural arrangement between two adjacent PSII complexes is altered in the absence of CP26 (Yakushevska et al. 2003; Vánská et al. 2025). Therefore, the loss of CP26 may affect interactions between LHCII and adjacent PSII complexes, which could lead to the pre-quenched state in *cp26*. Our results show that WT macro-organization of supercomplexes in the thylakoid membrane plays a key role in recovering PSII efficiency after high light exposure. F_q_′/F_m_′ values during high light illumination were similar, but F_v_′/F_m_′ values were decreased during dark recovery in lines carrying the CP26 mutation (Figure S2a). Although CP26 remains attached to the S-LHCII trimer and PSII core even at minimal antenna sizes (CS and C_2_S, Caffarri et al. 2009), these results indicate that the reduction of PSII efficiency due to the loss of CP26 is less pronounced under high light conditions as antenna size decreases to avoid overexcitation of PSII.

Previous research on the *cp26* SALK line used here (De Bianchi et al. 2008) suggested that the lack of CP26 might be complemented by upregulation of other minor antenna proteins. While we did see significant increases in some Lhcb proteins (Fig. 2f and S4c), these were not consistent between the single and double *cp26* mutants. This, together with the consistent observation of differences in measured chlorophyll fluorescence parameters, demonstrates that deficiency of CP26 is not functionally compensated for by any other LHCII protein, despite the structural similarity between different LHCII monomers (Croce and Van Amerongen 2011).

### Absence of PsbS and VDE exacerbates NPQ induction differences in *cp26* mutants

Experiments with *cp26* double mutants with contrasting levels of PsbS (Fig. 3) and VDE (Fig. 4a), as well as with leaf infiltration with the VDE inhibitor DTT (Fig. 4c), showed that the differences in NPQ during the slower phases of induction and relaxation between *cp26* mutants and their respective controls persisted and were extended to the fast phases of relaxation despite the absence of energy-dependent qE and zeaxanthin-dependent qZ. These results indicate that the effects of PsbS and VDE on NPQ are independent from the effect of CP26 mutation on NPQ induction (otherwise, NPQ traces would have converged throughout the measurements in the absence of either protein). Notably, the NPQ differences are more pronounced upon the alteration of qE components in contrast to the WT vs. *cp26* comparison (Fig. 2a). This may suggest that the phenotype of *cp26* becomes more visible under strong lumen acidification. These findings could be in line with *in vitro* results, which demonstrated PsbS-independent conformational changes in CP26 (Dall’Osto et al. 2005). However, in the results by Dall’Osto et al. (2005), these conformational changes required the presence of zeaxanthin, which we could not confirm here. Alternatively, removal of the qE and qZ contributions to NPQ in the *cp26* background may make effects on NPQ of structural PSII-LHCII reorganization (Ilíková et al. 2021; Vánská et al. 2025) more visible.

### The CP26 knockout effect on NPQ induction requires the proton gradient and depends on the protonation of lumen-exposed residues

Thylakoid lumen acidification through the build-up of a proton gradient across the thylakoid membrane is an important activator of NPQ. Evaluation of the steady-state proton gradient in *cp26* mutants over a range of light intensities (Fig. 6) did not show any impairment compared to the controls. This aligns with previous observations using 9-aminoacridine (De Bianchi et al. 2008) and demonstrates that differences in NPQ induction in *cp26* mutants are not explained by altered pmf formation or partitioning nor membrane proton conductivity. Consistent with the role of thylakoid lumen acidification on NPQ induction, leaves infiltrated with nigericin, which collapses the trans-membrane proton gradient, showed no difference in NPQ induction between *cp26* mutants and their controls. Importantly, the genotype pair of *npq4* mutants still retained NPQ differences in the mock treatment, when only PsbS is absent, but not upon chemical infiltrations, when both ΔpH and PsbS are missing (Fig. 5b). This was further corroborated by DCCD infiltration, which blocks protonatable protein residues in the thylakoid lumen (Ruban et al. 1992; Walters et al. 1996), again abolishing the effect of CP26 knockout on NPQ induction in all single and double mutants (Fig. 5b). Together, these findings suggest that the diminished NPQ induction effect in *cp26* depends on lumen acidification. Thus, while we cannot rule out direct involvement of CP26 on NPQ induction, the most likely explanation for these phenomena is that the altered thylakoid organization in the *cp26* mutant (Ilíková et al. 2021; Vánská et al. 2025), and altered stability of PSII supercomplex conformations in the absence of CP26 (Chen et al. 2018) leads to hyposensitivity of NPQ to ΔpH. As a result, the impact of structural PSII-LHCII supercomplex reorganization in *cp26* on NPQ induction is masked by removal of the proton gradient.

## Conclusions

The involvement of the minor antennae in NPQ is still debated (Xu et al. 2015; Dall’Osto et al. 2017; Townsend et al. 2018; Saccon et al. 2020; Ruban and Wilson 2021). There is evidence that a small proportion of overall NPQ may be associated with minor antennae quenching (Miloslavina et al. 2011; Dall’Osto et al. 2017), while the main quenching site appears to reside in detached major LHCII trimers (Nicol et al. 2019).

Here, we followed up on some long-standing hypotheses regarding the role of CP26 in NPQ. We confirmed that a CP26 knockout mutation leads to a sustained dark-quenched state (Nicol et al. 2019), which impairs photochemical efficiency in darkness. We also observed an effect of CP26 deletion on the slow phase of NPQ induction in single and double mutants and showed that this effect is neither dependent on the essential qE protein PsbS, consistent with Dall’Osto et al. (2005), nor on zeaxanthin, despite *in vitro* evidence that CP26 undergoes zeaxanthin-dependent conformational changes (Morosinotto et al. 2002; Dall’Osto et al. 2005). Instead, the dark quenched state and altered NPQ induction in *cp26* single and double mutants is most consistent with structural thylakoid reorganization.

## Materials and methods

### Plant material and growth conditions

All mutant lines were generated from the *Arabidopsis thaliana* wild-type Columbia-0 (WT Col-0). *Arabidopsis* mutant lines *cp26* (SALK-014869C, N656198), *npq4* (*PsbS*, N66021), and *npq1* (*VDE*, N3771) were obtained from the Nottingham Arabidopsis Stock Centre (NASC). The PsbS overexpression line L17 was generated by the Niyogi lab (Li et al. 2002a). Double mutants *cp26 npq4*, *cp26 L17,* and *cp26 npq1* were generated by cross-pollination and homozygous mutant lines were selected in the F2 generation by chlorophyll fluorescence screen under high light in the FluorCam imager (Photon Systems Instruments, Czech Republic) and by PCR screen using the primers LBb1.3/AT cp26_LP/AT cp26_RP (*cp26*), KN118/KN119 (*npq4*, Li et al. 2000), AT PsbS_2_S/AT scpl16_fw/AT scpl16_rv (L17), and KN75/KN76 (*npq1*, Niyogi et al. 1998) (Table S1). All mutants were confirmed on DNA and protein levels (Figure S1). The primers for L17 genotyping were developed by mapping the T-DNA insertion site for L17 to exon 11 of the serine carboxypeptidase-like 16 gene (*SCPL16*, AT3G12220) using TAIL-PCR (Singer and Burke 2003; see Figure S7). All plants were initially grown for 4 to 6 weeks under short-day-conditions (8 h light/16 h dark, 22 °C, 60% humidity, 150 µmol m^−2^ s^−1^) and then shifted to a growth cabinet with a 12-h light/dark cycle and similar conditions at least 2 days before experiments.

### Chlorophyll fluorescence measurements

Chlorophyll fluorescence was measured using the Dual-Klas-NIR instrument (Walz, Germany) coupled to a GFS-3000 measuring chamber and a LI-6800 gas exchange system (LI-COR, USA). Temperature was controlled at 25 °C via the measuring chamber, and CO_2_ (410 ppm) and humidity (60%) were controlled via the LI-6800 console. Whole plants were dark-acclimated for 75 min. Leaves clamped in the cuvette were exposed to two cycles of 20 min high light (1,000 µmol photons m^−2^ s^−1^ red actinic light) and 10 min dark relaxation. Chlorophyll fluorescence was measured using weak green PAM measuring light (540 nm). PSII efficiency parameters were determined by applying a saturating pulse (4,000 µmol photons m^−2^ s^−1^ red actinic light, 800 ms) directly after dark acclimation to measure the maximum PSII efficiency (F_v_/F_m_ = (F_m_−F_o_)/F_m_), followed by a 10-s dark period before switching on actinic light, and by applying further saturating pulses during high light exposure and dark relaxation to calculate non-photochemical quenching (NPQ = (F_m_−F_m_′)/F_m_′) and PSII operating efficiencies during illumination (F_q_′/F_m_′=(F_m_′-F′)/F_m_′) and dark relaxation (F_v_′/F_m_′=(F_m_′-F_o_′)/F_m_′). NPQ(T) was calculated from NPQ traces according to Tietz et al. (2017) (NPQ(T)= (4.88/((F_m_′/F_o_′)−1))−1), with F_o_′ being calculated according to Oxborough and Baker (1997) (F_o_′=F_o_/((F_v_/F_m_) + (F_o_/F_m_′))).

For infiltration with inhibitors, leaf segments of 1.5 cm × 1.5 cm were dark-acclimated on wet filter paper for 60 min, then vacuum-infiltrated in a syringe and briefly dried on filter paper (under dark conditions) and further dark-acclimated for 15 min inside the GFS-3000 measuring chamber before running a protocol of 10 min high light and 5 min dark relaxation with the same settings as described above. All inhibitors were dissolved in HEPES buffer (20 mM HEPES/KOH, pH 7.0). For removal of the proton gradient across the thylakoid membrane, leaves were infiltrated with 50 µM nigericin sodium salt (Sigma Aldrich; dissolved in ethanol). For blocking protonatable protein residues, leaves were infiltrated with 30 mM *N,N′*-dicyclohexylcarbodiimide (DCCD, Sigma Aldrich; dissolved in dimethylformamide, DMF). The VDE protein was inhibited by infiltration with 5 mM dithiothreitol (DTT, Sigma Aldrich). As a control, leaves were infiltrated with buffer only (mock infiltration).

Light curves were measured on intact leaves after 75 min dark acclimation. Light intensities were sequentially increased from low to high (0, 102, 185, 542, 853, and 1,302 µmol photons m^−2^ s^−1^) in steps of 5 min with a saturating pulse at the end of each step to calculate chlorophyll fluorescence parameters.

ETR(II) was calculated from F_q_′/F_m_′ values multiplied with the incident light intensity (PPFD), an assumed PSII/PSI ratio of 0.5 and genotype-specific absorbance factors (ETR(II) = F_q_′/F_m_′ * PPFD * 0.5 * Abs_total_). For *cp26* and WT plants, total light absorbance, *Abs_total_*, was estimated using a LI-1800 integrating sphere (LI-COR, USA) attached to a STS-VIS spectrometer (Ocean Optics, USA). *Abs_total_* was calculated by


$$Abs_{total}=\;(Abs_{630}\times \;0.9)+(Abs_{470}\;\times \;0.1),$$

where *Abs_630_* and *Abs_470_* are the absorbance estimates of red and blue light, respectively. These two values were adjusted to account for the proportions of each wavelength in the gas exchange instruments. *Arabidopsis* WT and *cp26* plants (n = 5) had *Abs_total_* values of 0.797 and 0.807, respectively.

### Gas exchange measurements

Net CO_2_ assimilation was measured using an open gas exchange system (LI-6800, LI-COR, USA). Following dark acclimation for 1 h, a fully-expanded leaf of intact 6- to 8-week-old plants was mounted into the measuring chamber of a fluorometer head (small aperture), dark acclimated for another 5 min, and subjected to two cycles of high light/dark periods (15 min 1,000 µmol photons m^−2^ s^−1^ and 15 min dark, logging every minute) or a light response curve with increasing PPFD and logging every 10 min (0, 50, 200, 500, 750, 1,000, and 1,500 µmol photons m^−2^ s^−1^). Cuvette conditions were controlled at 25 °C temperature, CO_2_ (410 ppm), and humidity (60%). IRGAs were matched just before the start of each measurement.

### Fluorescence lifetime snapshot measurements

Time-correlated single photon counting (TCSPC) was used to measure the initial chlorophyll fluorescence lifetimes after dark acclimation, as previously described (Steen et al. 2020). A Ti:sapphire oscillator (Coherent, Mira900f, 76 MHz) generated pulses at ∼808 nm and was frequency-doubled to ∼404 nm, which was used to excite the Soret band of chlorophyll *a*. This excitation beam was then divided by a beamsplitter, part of which was directed into a photodiode (Becker-Hickl, PHD-400) to provide SYNC signals. The other half of the excitation beam was then incident at an approximately 70° angle to the adaxial side of the leaf lamina. The excitation power was set to 1.0 mW, with a ∼600 µm beam diameter, which is enough to saturate the reaction centers. Fluorescence photons were detected by a microchannel plate (MCP)-photomultiplier tube (PMT)detector (Hamamatsu R3809U MCP-PMT) following a monochromator (HORIBA Jobin-Yvon; H-20), which was set to 680 nm, specifically detecting chlorophyll *a* Qy band fluorescence. A LabVIEW program was used to control a series of shutters, thereby coordinating the application of the excitation beam, the actinic light, and the detector. Within a 1 s total integration time of detection, a 0.2 s portion of the data showing the longest lifetime was selected for further data processing to ensure the saturation of the reaction centers (Sylak-Glassman et al. 2016). Each fluorescence decay profile was fitted with a bi-exponential decay function, and the amplitude-weighted average lifetime was calculated by:


$$\tau =\;\frac{\sum_{i}\;A_{i}\;\tau_{i}}{\sum_{i}\;A_{i}}$$

where $A_{i}$ and $\tau_{i}$ are the amplitudes and fluorescence lifetimes of the i^th^ fitting component, respectively.

### Electrochromic shift measurements

Steady state trans-thylakoid proton conductivity and proton motive force were estimated *in situ* on light-acclimated plants inside the growth cabinet with a hand-held MultispeQ device (Photosynq) using the protocol “Photosynthesis RIDES 2.0 EC 1000-no-open,” which estimates gH^+^, vH^+^, and ECSt parameters (Baker et al. 2007) from fast dark-induced relaxation kinetics.

Steady state trans-thylakoid proton gradient ΔpH and proton motive force (pmf) across a range of light intensities were estimated from slow dark-induced relaxation kinetics using the P515/P535 module for the Dual-KLAS-NIR (Walz, Germany) coupled to a GFS-3000 measuring chamber and a LI-6800 gas exchange system (LI-COR, USA). Temperature was controlled at 25 °C via the measuring chamber, and CO_2_ (410 ppm) and humidity (60%) were controlled via the LI-6800 console. Whole plants were dark-acclimated for 75 min. Leaves clamped in the cuvette were exposed to a light curve regime of 5 min light, followed by 5 min of dark over a range of increasing light intensities (73, 149, 479, 771, and 1,193 µmol photons m^−2^ s^−1^), and the P515 signal was detected with slow kinetics throughout the measurements. Upon light-to-dark transitions, pmf was calculated as the difference in P515 signal between the “light baseline” and the minimum value detected during the first seconds in the dark. P515 then increased again in the dark to a “dark baseline,” and ΔΨ was calculated as the difference between both baselines (Cruz et al. 2001). ΔpH was calculated as ΔpH = pmf-ΔΨ, and the proportion of ΔpH to pmf was plotted.

### Blue-native polyacrylamide gel electrophoresis (BN-PAGE) and western blotting

Thylakoid membrane proteins were isolated from 8-week-old plants according to Järvi et al. (2011). One plant per genotype was either treated with 1,000 µmol photons m^−2^ s^−1^ white high light or dark-acclimated for 1 h, with three biological replicates each. Fresh whole rosettes were ground in 20 ml ice-cold grinding buffer either under high light or in darkness and filtered through two layers of Miracloth (Sigma Aldrich). Extracts were further treated on ice and in dim light by several centrifugation steps and resuspension of pellets in shock and storage buffers. All buffers contained 10 mM NaF to preserve protein phosphorylations. After the final resuspension step in 200 µl of storage buffer, the chlorophyll *a* content was determined in 100% methanol according to Porra et al. (1989), using absorption values at wavelengths 652.0, 665.2, and 750 nm for the equation: chlorophyll *a* [μg/ml] = 16.29×(A_665.2_ − A_750_) – 8.54×(A_652.0_ − A_750_).

For BN-PAGE analysis, extracts were resuspended with freshly prepared ice-cold ACA buffer (containing 10 mM NaF) to a concentration of 1 mg chlorophyll/ml. Aliquots of 5 µg chlorophyll were resuspended with an equal volume of 4% digitonin (Sigma Aldrich) in ACA buffer (final concentration of 2% digitonin), and thylakoid membrane proteins were solubilized at room temperature for 10 min on a shaker. Afterwards, samples were spun down for 20 min at 18,000*xg* at 4 °C to pellet insolubilized membranes. The supernatant was mixed carefully with 1/10 of the volume with sample buffer, loaded onto a 3 to 12% Bis-Tris NativePAGE gradient gel (Invitrogen), and the electrophoresis was run on ice for five hours with increasing voltage (75 to 150 V).

Protein abundance was compared between genotypes using thylakoid samples in western blot analyses. Samples equivalent to 1 µg chlorophyll were solubilized with 2× sample buffer (Laemmli buffer + 6 M urea + β-mercaptoethanol + Bromophenol blue) for 5 min at 75 °C and loaded onto 12% Mini-PROTEAN® TGX™ Precast Gels (Bio-Rad). After protein separation, proteins were blotted onto PVDF membranes with a Trans-Blot Turbo Transfer System (Bio-Rad). Blots were washed in TBS, blocked with 5% milk in T-TBS for 1 h, washed twice with T-TBS, and incubated overnight with primary antibodies in 1% milk in T-TBS, shaking at 4 °C (dilutions according to manufacturer's instructions). The day after, blots were washed four times with T-TBS, incubated in secondary antibody (goat anti-rabbit IgG, HRP conjugated, dilution = 1:12,500 in 1% milk in T-TBS) for 1 h, and washed twice with T-TBS and three times with TBS. For the detection of protein bands, blots were incubated in ECL solution for 5 min and imaged with a G:Box Chemi XRQ system (Syngene). Image analysis and quantification of bands were performed in ImageJ, using a dilution series of WT samples as a standard on each blot. All primary and secondary antibodies were obtained from Agrisera/Newmarket Scientific (Sweden/UK).

### Statistical analyses

All statistical analyses were performed in R (version 4.4.0 and higher) using the packages “rstatix,” “ggpubr,” and “car.” Time course datasets were first tested for extreme outliers and normal distribution of data points for each genotype at each time point (Shapiro-Wilk test). A two-way repeated measures ANOVA was conducted to determine the effects of genotype and time on NPQ, F_q_′/F_m_′, F_v_′/F_m_′, ETR(II), A_net,_ and NPQ(T). If a significant interaction effect (Genotype:time/PPFD) was detected, a post-hoc *t*-test with Bonferroni-adjusted *P*-values was performed to obtain the genotype effect between *cp26* mutants and their respective controls at each time point (Supplementary Dataset S1).

For datasets presented in boxplots, a Student's *t*-test was used to compare the means of two genotypes (*cp26* mutants vs. their controls) using the ggplot2 package. Before that, Levene's test was used to check for equality of group variances.

## Supplementary Material

kiag207_Supplementary_Data

## Data Availability

The data underlying this article are available in the article and in its online Supplementary material.
