# Supplementary material for: CP26 is not involved in qE- or qZ-type non-photochemical quenching in Arabidopsis
Source: Plant Physiol. 2026 Apr 16;201(1):kiag207. doi: 10.1093/plphys/kiag207 (PMC13191597; doi:10.1093/plphys/kiag207)
Supplement: kiag207_Supplementary_Data [file kiag207_supplementary_data.zip › Supplementary Data.pdf]

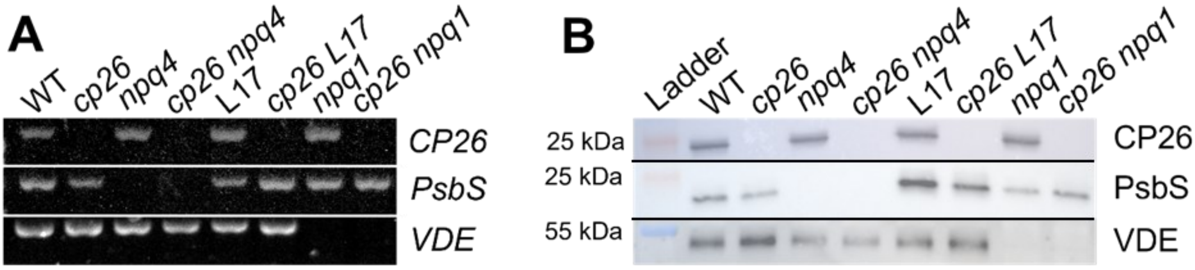

Supplementary Figure S1: Molecular confirmation of *Arabidopsis* mutants used in this study.

A) DNA confirmation with PCR of *CP26*, Photosystem II Subunit S (*PsbS*) and violaxanthin de-epoxidase (*VDE*) genes. **B)** Western blot results using CP26, PsbS and VDE antibodies (1 µg chlorophyll of thylakoid membrane protein extracts loaded).

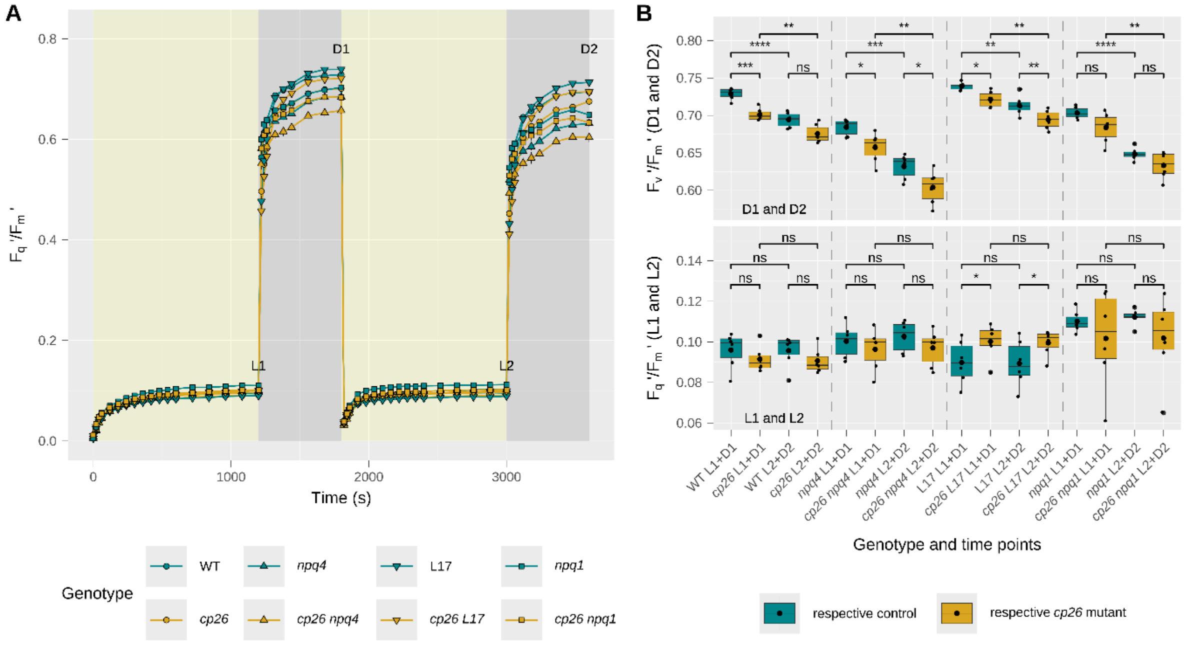

Supplementary Figure S2: Photosystem II (PSII) quantum efficiencies in *cp26* and double mutants with contrasting PSII subunit S (PsbS, knockout = *npq4* and overexpression = L17) and Violaxanthin de-epoxidase (VDE, *npq1*) alleles.

A) PSII quantum yields, F_q'/F_m', measured upon high light induction (1000 µmol photons m^{-2} s^{-1} for 20 min, yellow background), followed by 10 min of dark relaxation (dark grey background), in two consecutive cycles. **B)** Maximum F_v'/F_m' values (top panel, measured at the end of the two dark phases and annotated as D1 and D2) and maximum F_q'/F_m' values (bottom panel, measured at the end of the two high light phases and annotated as L1 and L2).

Line plots show mean values and error bars indicate the standard error of the mean for control genotypes (wild-type WT, *npq4*, L17 and *npq1*; green lines and circles, upwards-pointing triangles, downwards-pointing triangles, and squares, respectively) and *cp26* mutants (*cp26*, *cp26 npq4*, *cp26 L17* and *cp26 npq1*; yellow lines and circles, upwards-pointing triangles, downwards-pointing triangles, and squares, respectively). Boxplots contain individual data points (black dots), the median (black line inside the box) and the mean (larger black dot inside the box). Boxes show upper and lower quartiles, whiskers show the 1.5x interquartile range, points outside whiskers show outliers. Data were collected from six biological replicates (n=6). Significant differences between control genotypes (green boxes) and *cp26* mutants (yellow boxes) are indicated by asterisks (two-way repeated measures ANOVA within D1 & D2 and L1 & L2 maximum values). To compare D1 & D2 and L1 & L2 time points between genotypes, a Student's t-test was applied. Significance levels: * = p<0.05, ** = p<0.01, *** = p<0.001 and **** = p<0.0001.

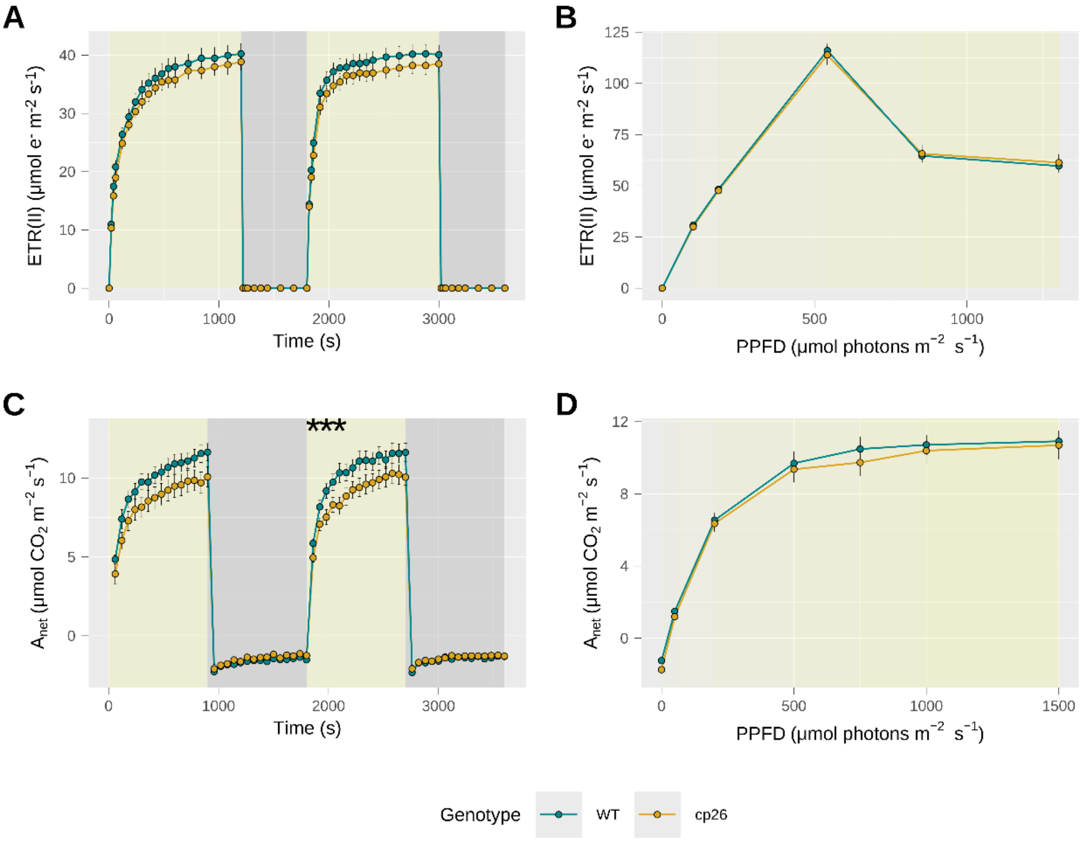

Supplementary Figure S3: Photosystem II (PSII) electron transfer and net CO_2 assimilation rates in *cp26*.

The electron transfer rate in PSII, ETR(II), was determined from F_q'/F_m' data (Dual-Klas-NIR, Walz) in Figs. S2A and 2C during high light induction (1000 µmol photons m^{-2} s^{-1} for 20 min) and dark relaxation (10 min) in two consecutive cycles (**A**), and in a light response curve (**B**), respectively. Net CO_2 assimilation, A_{net}, was similarly measured in two cycles of high light/dark periods (15 min each) (**C**), and in a light response curve at increasing levels of photosynthetic photon flux density (PPFD, **D**), using LI-6800s (LI-COR).

Line plots show mean values from six biological replicates (n=6) for ETR(II), and 9-16 biological replicates (n=9 in **C**, and n=16 in **D**) for A_{net}. Error bars indicate the standard error of the mean. The asterisk represents significant differences between *Arabidopsis* wild-type (WT, green lines and circles) and *cp26* mutant (yellow lines and circles) at each measurement point (two-way repeated measures ANOVA with following post-hoc t-test with Bonferroni correction). In **A**, **B** and **D**, Genotype:time and Genotype:PPFD interaction effects were not significant ($p>0.05$). Significance level: * = $p<0.05$.

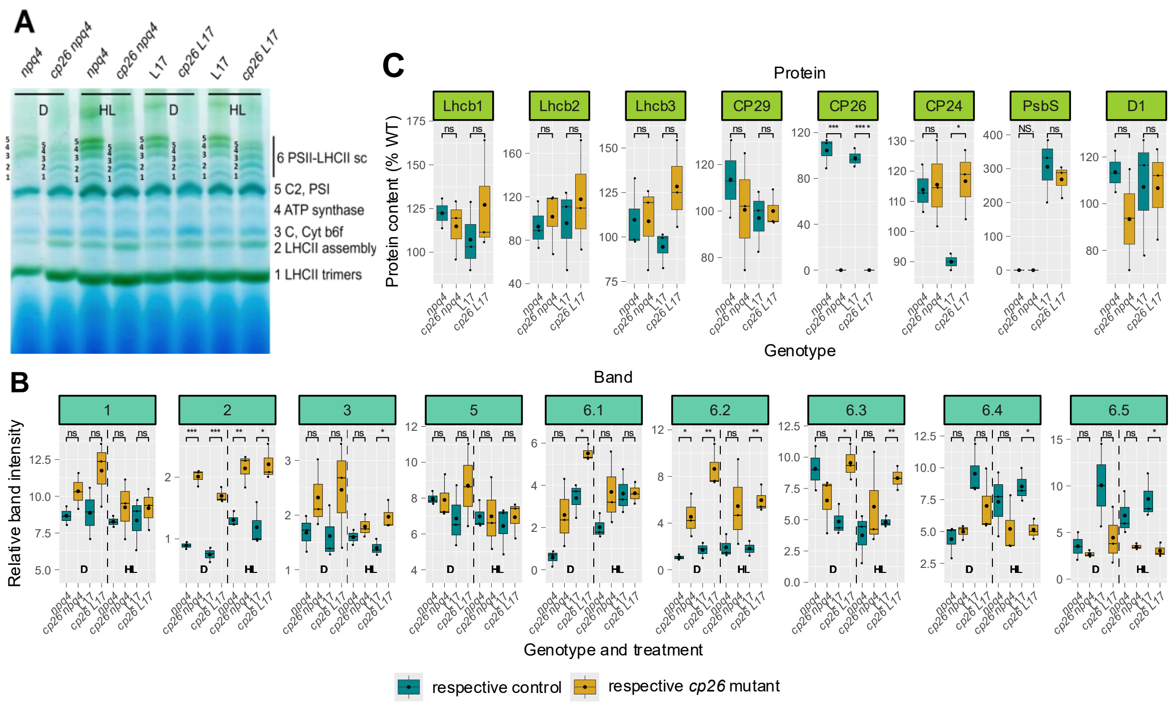

Supplementary Figure S4: Thylakoid membrane protein analyses of *cp26* mutants crossed with contrasting Photosystem II subunit S (PsbS) alleles (knockout = *npq4* and overexpression = L17).

A) Blue Native-Polyacrylamide Gel Electrophoresis gel of dark (D) and high light (HL)-treated samples from *cp26* double mutants either lacking PsbS (*cp26 npq4*) or overexpressing PsbS (*cp26 L17*) and their respective controls *npq4* and L17; representative image from three biological replicates (n=3). Labelled bands correspond to: 1) Light-harvesting complex II (LHCII) trimers, 2) LHCII assembly, 3) PSII monomers (C) and Cytochrome b6f, 4) Adenosine Triphosphate synthase, 5) PSII dimers and PSI, 6) PSII-LHCII supercomplexes; according to Järvi *et al.* (2011). **B)** Band intensities corresponding to the bands in **A**, determined with ImageJ and normalized to the intensity of band 4. **C)** Western blot analyses with ImageJ from three biological replicates (n=3) to quantify the abundance of thylakoid membrane proteins relative to the wild-type (WT, see Fig. 2F).

Boxplots (n=3) contain individual data points (black dots), the median (black line inside the box) and the mean (larger black dot inside the box). Boxes show upper and lower quartiles, whiskers show the data range. Significant differences between single mutants (*npq4* and L17, green boxes) and *cp26* mutants (yellow boxes) were determined using Student’s t-test. Significance levels: ns/NS. = no significance and * = p<0.05, ** = p<0.01, *** = p<0.001 and **** = p<0.0001.

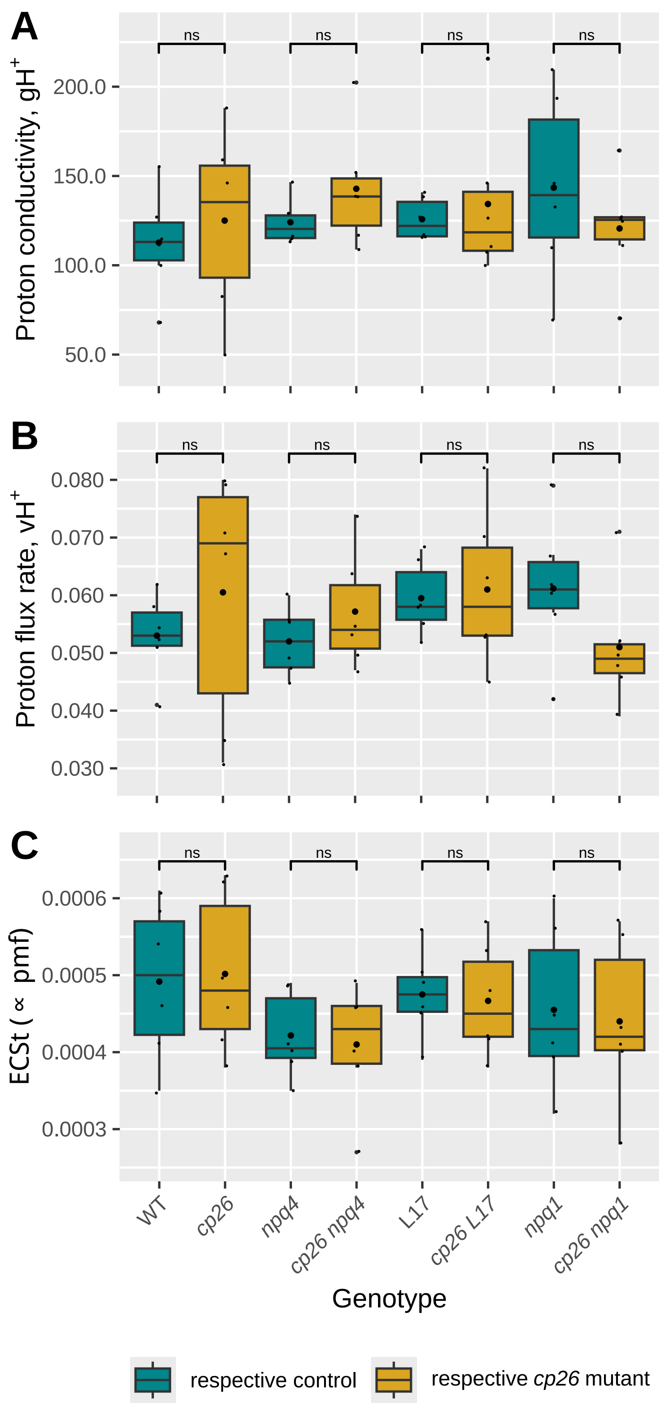

Supplementary Figure S5: Evaluation of the proton gradient across the thylakoid membrane.

A) ATP synthase proton conductivity, gH^+, **B)** steady-state proton flux rate, vH^+, and **C)** ECSt (electrochromic shift signal during light-to-dark transition; proportional to trans-thylakoid proton motive force, pmf, Baker *et al.*, 2007) were measured from six biological replicates (n=6) using the MultispeQ (Photosynq) and a modified RIDES protocol with a light intensity of 1000 µmol photons m^{-2} s^{-1}.

Boxplots contain individual data points (black dots), the median (black line inside the box) and the mean (larger black dot inside the box). Boxes show upper and lower quartiles, whiskers show the 1.5x interquartile range, points outside whiskers show outliers. Significant differences between *cp26* mutants (*cp26*, *cp26 npq4*, *cp26 L17* and *cp26 npq1*; yellow boxes) and their respective controls (wild-type WT, *npq4*, L17 and *npq1*; green boxes) were assessed using Student's t-test (significance levels: ns = no significance).

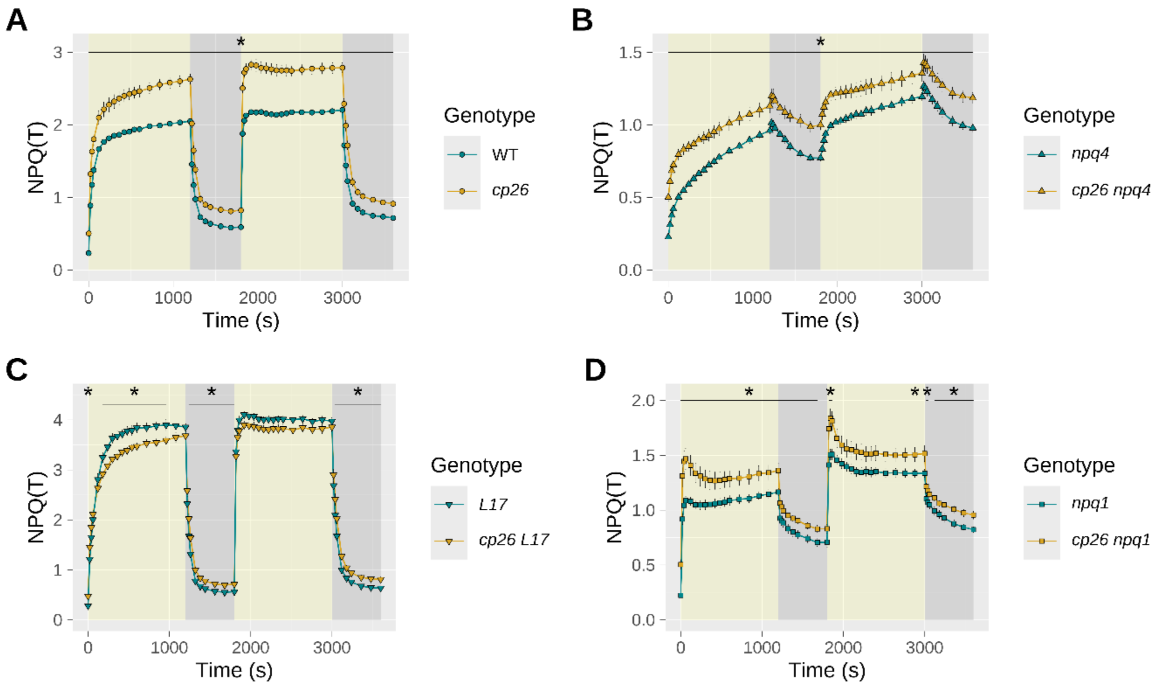

Supplementary Figure S6: NPQ(T), calculated from Non-photochemical quenching (NPQ) measurements in Figs. 2A, 3, and 4A according to Tietz *et al*. (2017), assuming F_v/F_m values of 0.83 for all genotypes.

NPQ(T) during high light (1000 µmol photons m^{-2} s^{-1} for 20 min; yellow background) and dark phases (10 min; dark grey background) in two consecutive cycles. **A)** wild-type (WT) and *cp26*, **B)** *npq4* and *cp26 npq4*, **C)** L17 and *cp26 L17*, **D)** *npq1* and *cp26 npq1*.

Data were collected from six biological replicates (n=6). Line plots show mean values with error bars indicating the standard error of the mean. The asterisk designates significant differences between *cp26* mutants (yellow symbols) and their respective controls (green symbols) (two-way repeated measures ANOVA with post-hoc t-test with Bonferroni correction). Significance level: * = p<0.05.

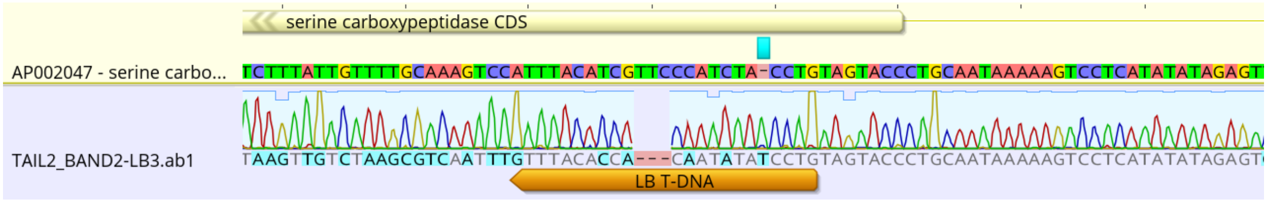

Supplementary Figure S7: Mapping the L17 T-DNA insertion site.

The sequenced PCR product resulting from TAIL-PCR was mapped to the coding sequence of exon 11 of the serine carboxypeptidase-like 16 gene (*SCPL16*, AT3G12220) using Geneious Prime. The left border T-DNA repeat sequence in the TAIL-PCR product (LB T-DNA) and the putative T-DNA insertion site in the *SCPL16* gene (light turquoise rectangle) are highlighted.

SupplementaryTable S1: Primers used in this study.

Primer name	Primer sequence
LBb1.3	5'-ATTTTGCCGATTTCGGAAC-3'
AT cp26_LP	5'-CGCCACTAGTGATAAAATCGC-3'
AT cp26_RP	5'-TAAACGGTGAAGTTGCTGGAG-3'
KN118 (*PsbS*)	5'-TCCTTCTCTCATCCTCAGAAA-3'
KN119 (*PsbS*)	5'-CAACATGAAGAGAAGGTCACA-3'
AT psbs_2_S	5'-TCGTTGGTCGTGTTGCTATG-3'
AT scpl16_fw	5'-TTATAGTCTAACGTGTGCCC-3'
AT scpl16_rv	5'-CGATGAAGAGAACTTGTGGA-3'
KN75 (*VDE*)	5'-GGGGAAGATTAGATAGTGTGA-3'
KN76 (*VDE*)	5'-TTACTTTATATGAACCGAACA-3'
