# Supplementary material for: CP26 is not involved in qE- or qZ-type non-photochemical quenching in Arabidopsis
Source: Plant Physiol. 2026 Apr 16;201(1):kiag207. doi: 10.1093/plphys/kiag207 (PMC13191597; doi:10.1093/plphys/kiag207)
Supplement: kiag207_Supplementary_Data [file kiag207_supplementary_data.zip › Supplementary File S1.docx]

**A
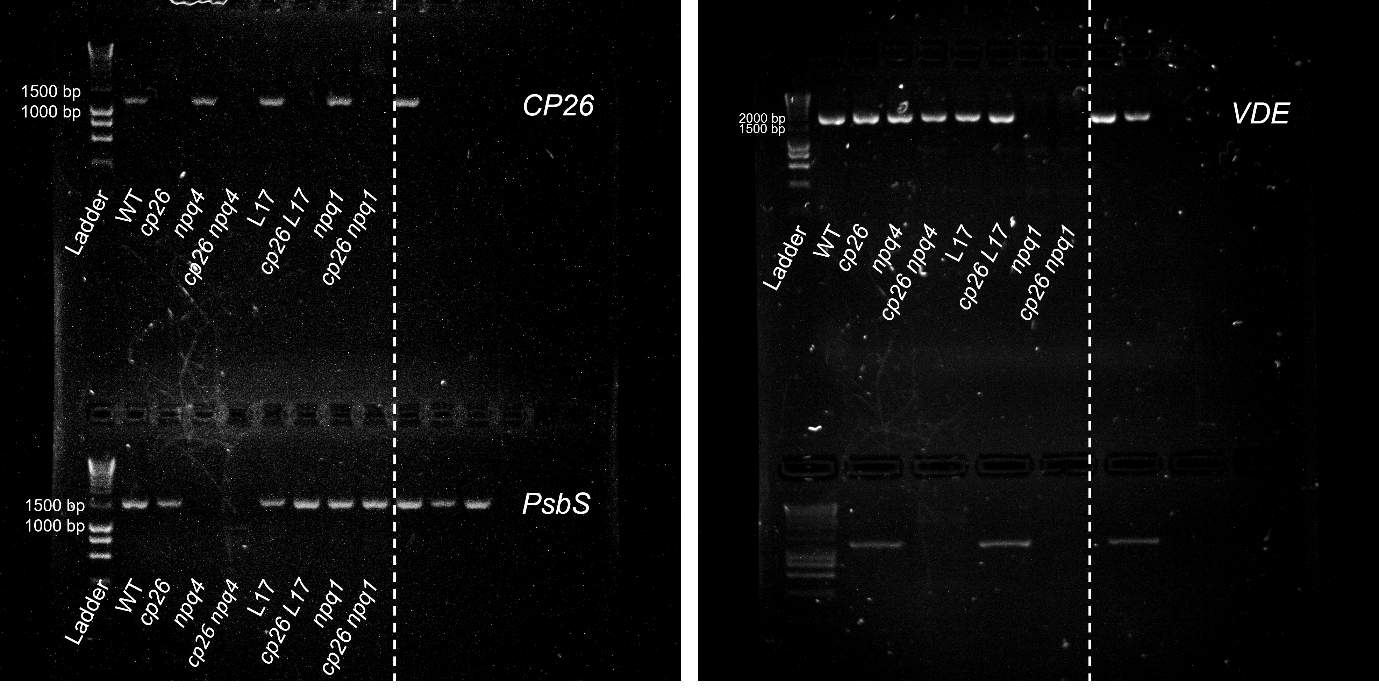
**

**B**


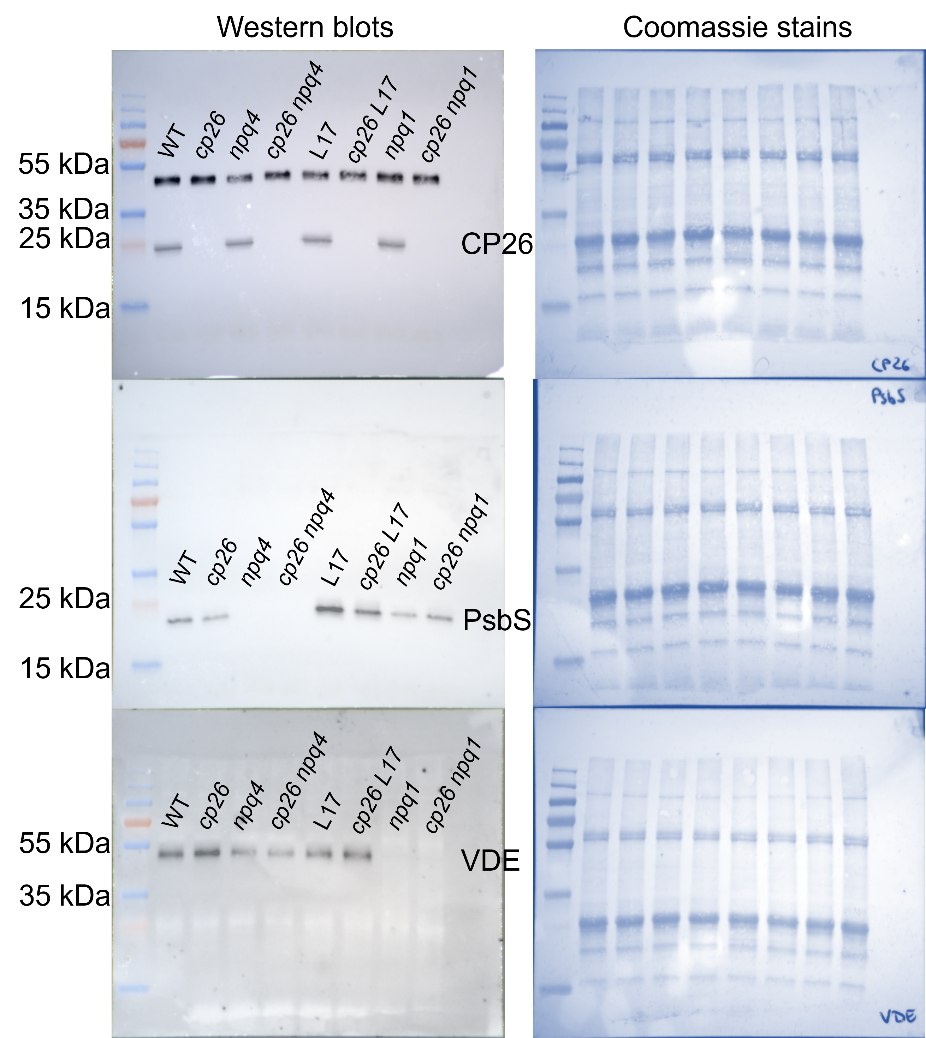


**Fig. S1:** Original image files of DNA agarose gels **(A)** and Western blot membranes before and after Coomassie staining **(B)**.

**D**  **
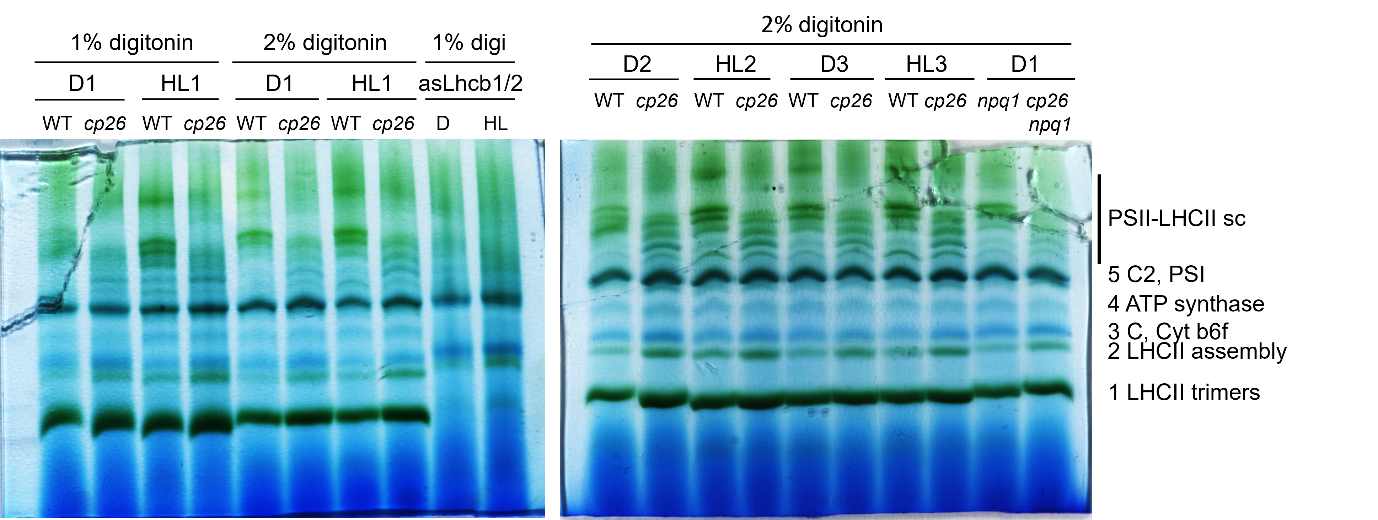
**

**Fig. 2: D)** Original BN-PAGE gels containing thylakoid extracts from three biological replicates of dark-acclimated (D) and high light-treated (HL) WT and *cp26* plants. Two concentrations of the detergent digitonin were tested (1% vs. 2%) and the antisense mutant asLhcb2-12 (NASC ID: N6363; Andersson *et al*., 2003, https://doi.org/10.1046/j.1365-313X.2003.01811.x), which lacks Lhcb1 and Lhcb2 proteins, was used to confirm the identities of bands 1 and 2 as LHCII subunits.


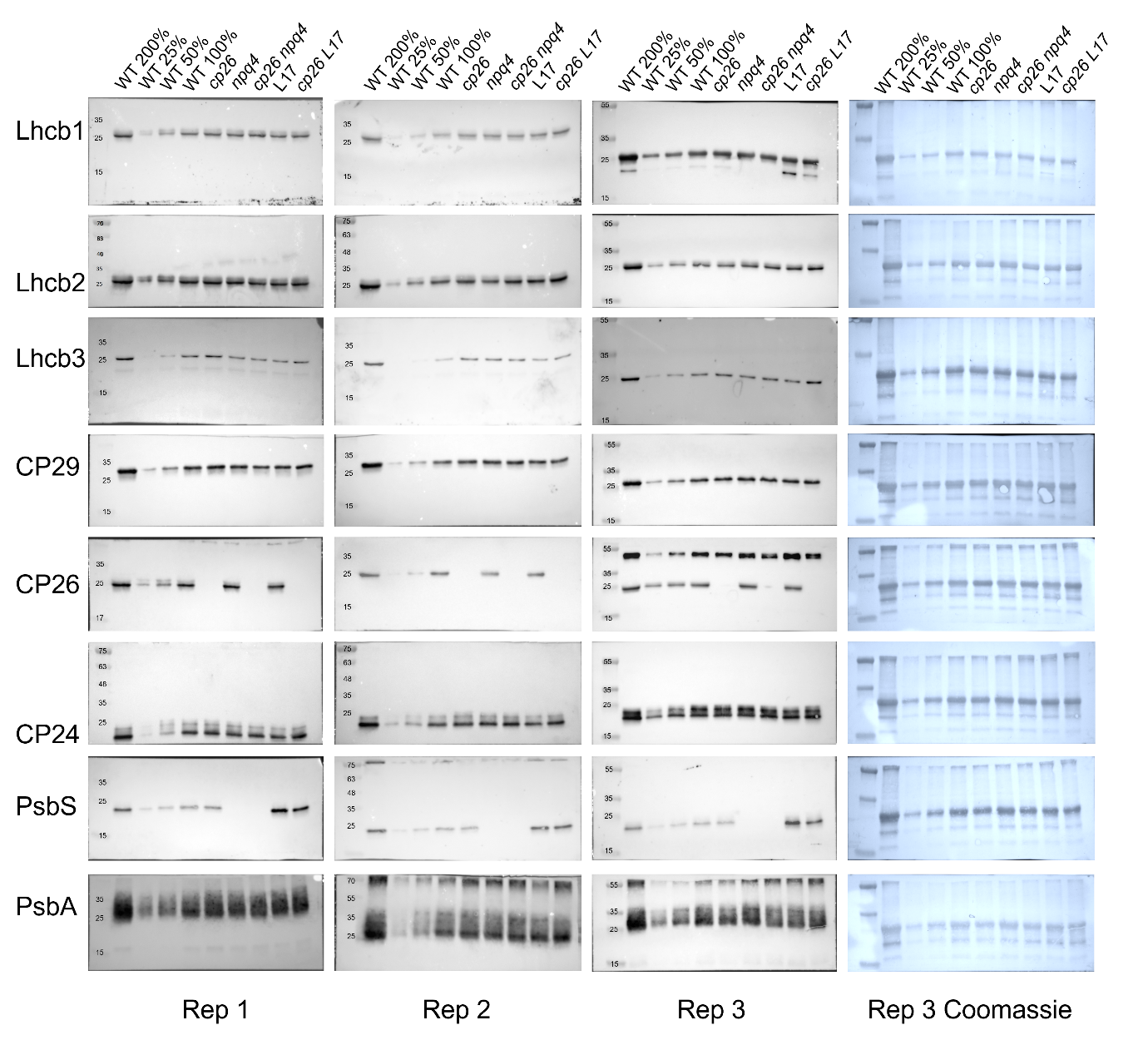


**Fig. 2F** and **Fig. S4C**: Original Western blot images from three biological replicates from high light-treated plants with Coomassie stained membranes for Rep 3 as a loading control.

**A**


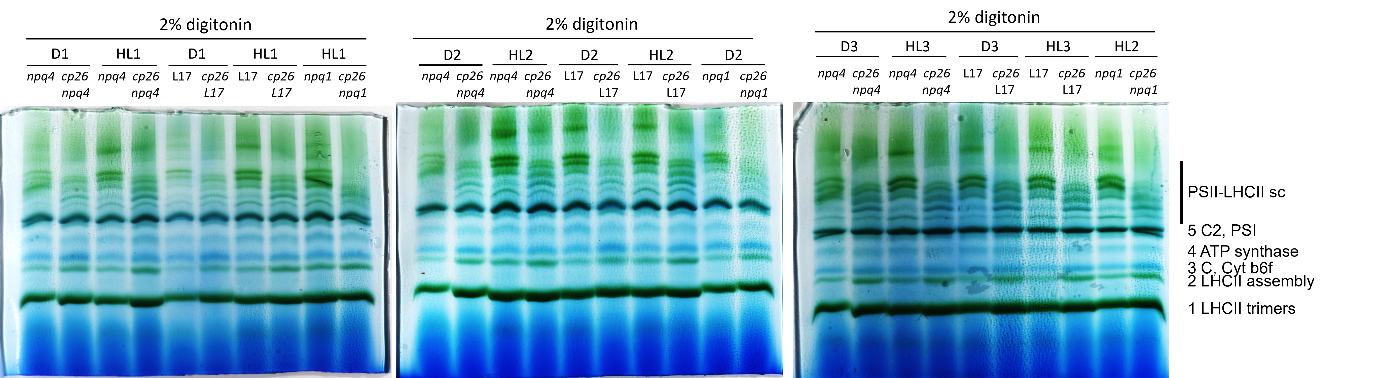


**Fig. S4: A)** Original BN-PAGE gels containing thylakoid extracts from three biological replicates of dark-acclimated (D) and high light-treated (HL) *npq4* and L17 single and double mutants (replicates of *npq1* mutants not included in analysis).


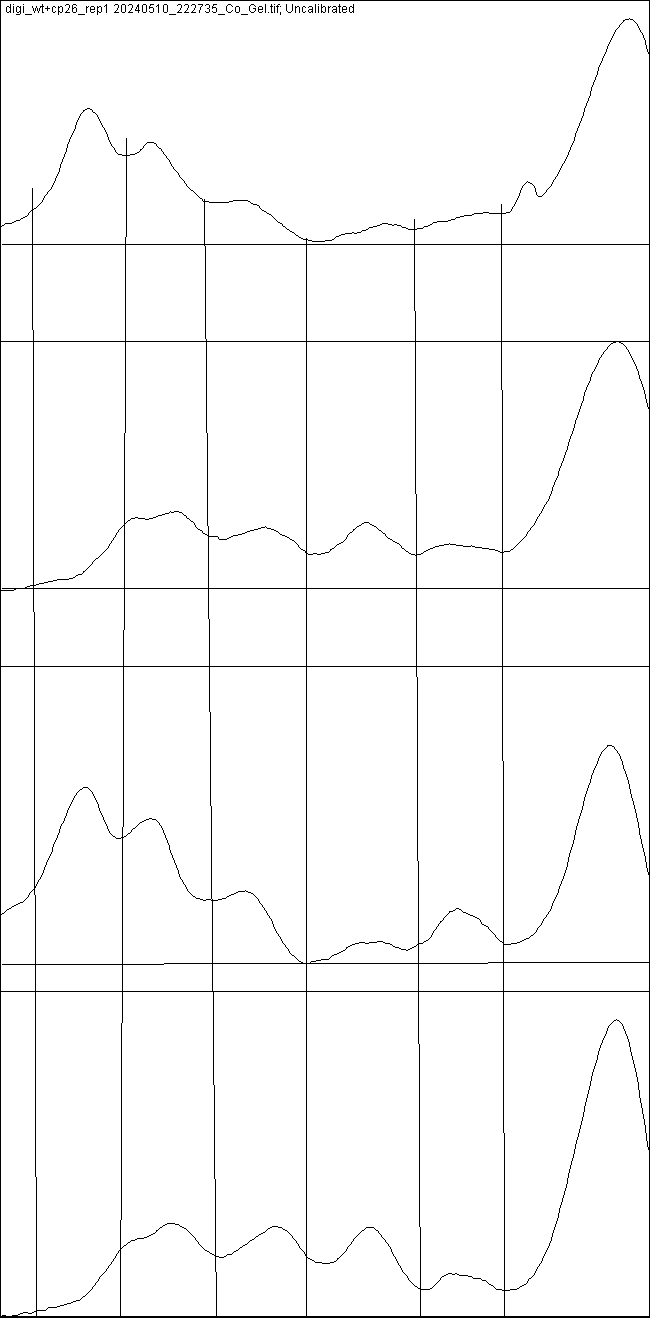

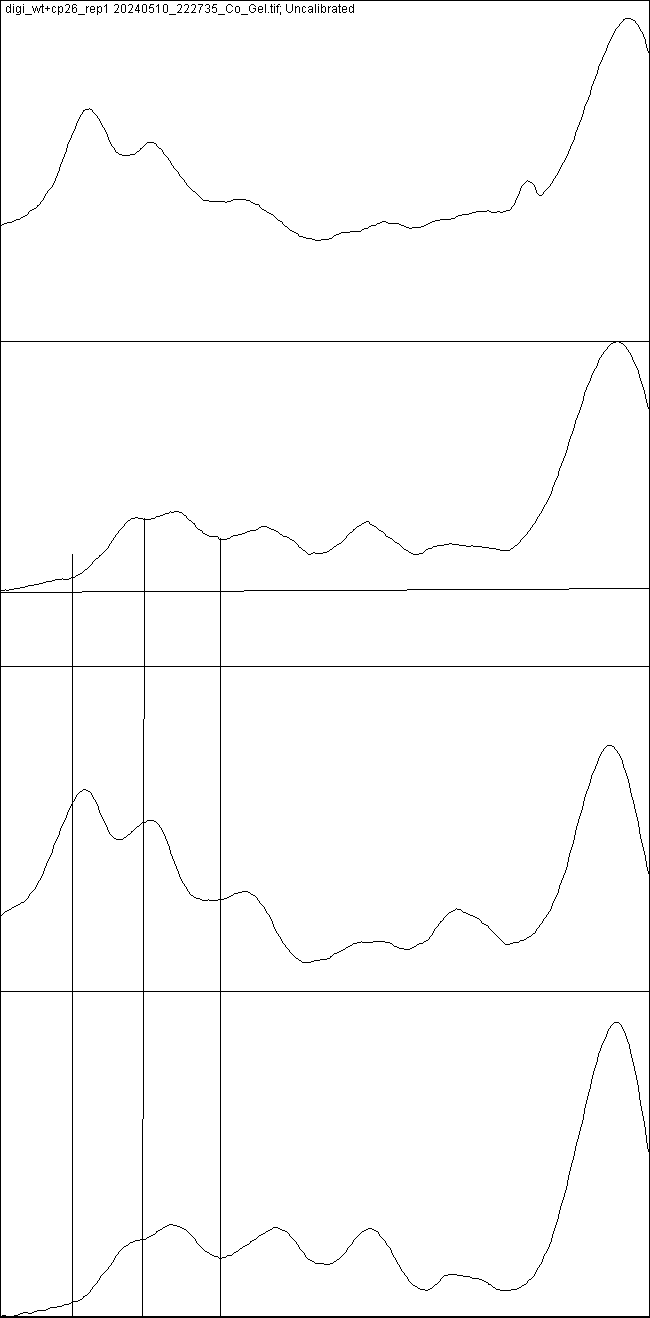


Band

6.5

Band

6.4

*cp26*, HL sample, Rep1

WT, HL sample, Rep1

WT, Dark sample, Rep1

Band

6.5

Band

6.4

Band

6.3

Band

6.2

Band

6.1

*cp26*, Dark sample, Rep1

Band

6.3

Band

6.2

Band

6.1

**Fig. 2D/E:** Example of WT and *cp26* ImageJ densitometry analyses of the PSII-LHCII supercomplex bands at the top of BN-PAGE gels (bands 6.1-6.5), demonstrating a shift of the top two bands, 6.5 and 6.4, in *cp26* compared to the WT.
